# Optomechanical Hot-Spots in Metallic Nanorod–Polymer Nanocomposites

**DOI:** 10.1021/acsnano.2c06673

**Published:** 2022-12-07

**Authors:** Thomas Vasileiadis, Adnane Noual, Yuchen Wang, Bartlomiej Graczykowski, Bahram Djafari-Rouhani, Shu Yang, George Fytas

**Affiliations:** † Faculty of Physics, Adam Mickiewicz University, 61-614 Poznan, Poland; ‡ LPMR, Département de Physique, Faculté des Sciences, Université Mohammed Premier, Oujda, 60000, Morocco; § Department of Materials Science and Engineering, University of Pennsylvania, 3231 Walnut Street, Philadelphia, Pennsylvania 19104, United States; ∥ Max Planck Institute for Polymer Research, 55128 Mainz, Germany; ⊥ Département de Physique, Institut d’Electronique de Microélectonique et de Nanotechnologie, UMR CNRS 8520, Université de Lille, Villeneuve d’Ascq, 59655, France

**Keywords:** optomechanics, Brillouin light scattering, plasmonic enhancement, elastic vibrations, nanorods, polymer nanocomposites

## Abstract

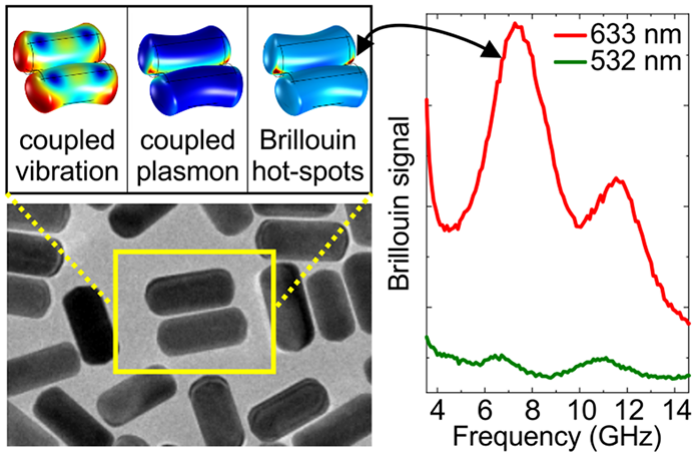

Plasmonic coupling between adjacent metallic nanoparticles can be exploited for acousto-plasmonics, single-molecule sensing, and photochemistry. Light absorption or electron probes can be used to study plasmons and their interactions, but their use is challenging for disordered systems and colloids dispersed in insulating matrices. Here, we investigate the effect of plasmonic coupling on optomechanics with Brillouin light spectroscopy (BLS) in a prototypical metal–polymer nanocomposite, gold nanorods (Au NRs) in polyvinyl alcohol. The intensity of the light inelastically scattered on thermal phonons captured by BLS is strongly affected by the wavelength of the probing light. When light is resonant with the transverse plasmons, BLS reveals mostly the normal vibrational modes of single NRs. For lower energy off-resonant light, BLS is dominated by coupled bending modes of NR dimers. The experimental results, supported by optomechanical calculations, document plasmonically enhanced BLS and reveal energy-dependent confinement of coupled plasmons close to the tips of NR dimers, generating BLS hot-spots. Our work establishes BLS as an optomechanical probe of plasmons and promotes nanorod–soft matter nanocomposites for acousto-plasmonic applications.

Metallic nanostructures hosting charge oscillations with strong electromagnetic (EM) near-fields, termed plasmons, have myriads of applications, including sensing,^1^ photochemistry,^2^ and solar energy harvesting.^3^ The energy and symmetry of plasmons are strongly dependent on the composition, shape, and size of the metallic nanostructures, as well as the optical properties of their environment. The connection between static structure and plasmonic functionalities has been exhaustively studied over several decades. More recent efforts are concentrated on the effect of dynamical morphological changes on plasmons, which can be induced, for instance, with confined acoustic vibrations in the gigahertz (GHz) frequency range.^4^ The emerging field of acousto-plasmonics aims to exploit acoustic vibrations and plasmons for sensing and signal-processing applications.^5−8^ So far, plasmons were mostly utilized for efficient generation of propagating acoustic wavepackets, i.e., as efficient acoustic wave sources.^7,8^ Given recent developments in all-optical generation and sensing of acoustic waves,^9^ an exciting prospect is to realize plasmonically enhanced acoustic detectors.

The complex interplay between light, plasmons, and acoustic vibrations can be understood through the theory of optomechanics. The applicability of optomechanical principles to the single-molecule level provides a theoretical description of surface-enhanced Raman scattering (SERS).^10−12^ However, while SERS can exhibit plasmonic enhancement factors by an order of 10^8^ for terahertz molecular vibrations,^1^ the plasmonic enhancement of Brillouin light spectroscopy (BLS) of acoustic vibrations remains limited. Yet, it is known that plasmons can modify the inelastic scattering selection rules leading to the appearance of new BLS peaks.^13−15^ Previous BLS studies of plasmonic systems have mostly focused on colloidal dispersions of nanospheres.^13,14,16^ Only recently was this work extended to nanoparticles with broken rotational symmetry such as nanorods^15^ (NRs) on a Si wafer, providing evidence of plasmonically modified BLS selection rules for the normal modes of a single NR. Others have observed the enrichment of vibrational spectra of individual gold nanoparticles on thin SiO_2_ substrates due to the highly anisotropic electromagnetic fields of plasmons.^17^ However, the current optomechanical models provide only a qualitative explanation of the BLS spectra of nonspherical plasmonic nano-objects.^15^ Moreover, the bulk of the studies on the vibrational properties of NRs used time-domain pump–probe methods,^18−23^ which can only capture the first extensional and breathing modes, as well as electron–lattice interactions.

In this work, we have used BLS as an optomechanical probe of plasmonic and vibrational interactions between metallic NRs dispersed in a soft polymer matrix. Most acousto-plasmonic systems examined so far consisted of stiff metal-decorated surfaces hosting surface acoustic waves,^7,8^ particle-on-mirror geometries,^24^ molecular-like structures on surfaces,^25^ and self-assembled colloids.^5^ Metal–soft matter nanocomposites can also be promising materials for acousto-plasmonic devices with a wide range of tunability, especially using anisotropic metal NRs. In principle, soft polymer materials can host propagating acoustic waves that modulate the plasmonic coupling between adjacent nanostructures. Moreover, embedding nanostructures into bulk materials can lead to modified phonon transport properties, which can then be studied with BLS.^26^ Self-assembled nanostructures dispersed in polymers offer temperature- and pressure-tunability,^27^ photothermal^28,29^ and photoactuating properties,^30^ and the ability to construct flexible devices and to use a multifarious toolbox for nanofabrication.^31^

As a model system, we studied gold nanorods (Au NRs) of various aspect ratios in poly(vinyl alcohol) (PVA) at 1 vol %. The PVA has been found to provide a more homogeneous dispersion of the Au NRs compared to other polymers such as poly(vinyl pyrrolidone) (PVP), based on their extinction spectra. By studying these samples with BLS, we aim to advance the field by (i) going beyond spherical objects,^13,14^ single NR normal modes,^15^ and quasi-translational modes^32^ to show how plasmonic coupling enables the BLS detection of bending-like coupling modes in a system with broken rotational symmetry, (ii) quantifying the wavelength-dependent plasmonic enhancement of BLS, and (iii) developing experimentally constrained computational methods for studying the optomechanical (OM) coupling in complex nanostructures that do not rely on spherical^33^ or cylindrical symmetry.^15^

## Results and Discussion

### Gold Nanorod Morphology

**Figure 1:**
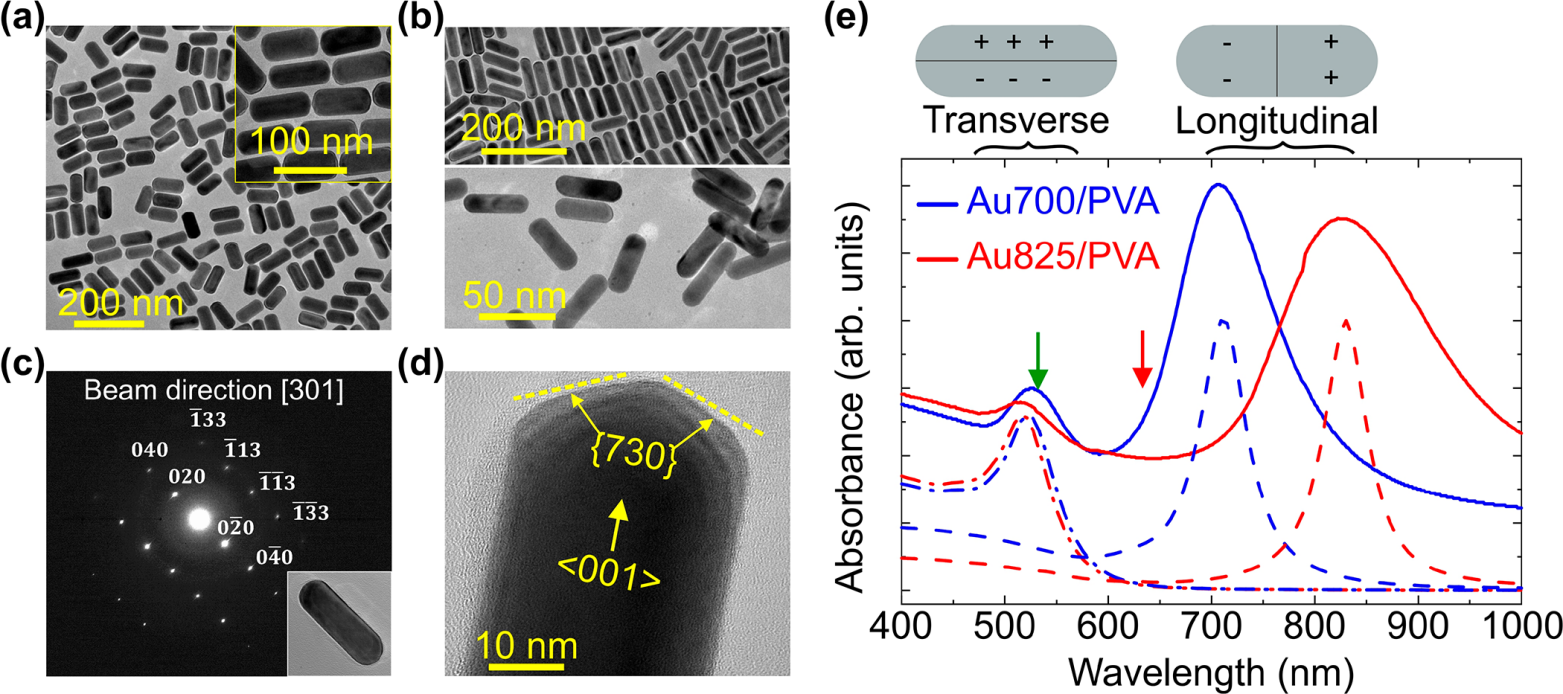
Structure and extinction spectrum of Au NRs. (a) TEM image of Au700 NRs and a higher magnification image shown as inset. (b) TEM images of Au825 (up) and Au800 (down) drop casted from a water suspension on standard TEM grids. (c) TEM diffraction pattern of a Au825 NR (inset) verifying its single-crystalline structure. (d) HR-TEM image and illustration of the ⟨001⟩ crystal growth direction and the {730} surface facets of a single Au700 NR. (e) Experimental extinction spectra (solid lines) of Au700 (blue) and Au825 (red) dispersed in polyvinyl alcohol (PVA), and FEM calculations of the extinction cross-section of transverse (dashed dot) and longitudinal (dashed) single NR plasmons. For the calculated spectra, the terms “longitudinal” or “transverse” refer to the polarization of the incident light being parallel or normal to the ⟨001⟩ axis of the Au NRs, respectively. The intensities of all curves have been scaled for comparison. The arrows correspond to the 532 nm (green) and 633 nm (red) laser wavelengths used for the BLS measurements.

We prepared and characterized (see Methods and Supporting Information S1) three different types of Au NRs dispersed in PVA: Au700 with length 82.1 ± 4.2 nm and diameter 35.7 ± 2.3 nm, Au825 with length 81.1 ± 5.7 nm and diameter 24.1 ± 1.3 nm, and Au800 with length 42.5 ± 4.5 nm and diameter 12.2 ± 2.9 nm. The shape and crystal structures of the Au NRs, drop-cast from an aqueous solution on transmission electron microscopy (TEM) grids, are shown in Figure 1a for Au700 and Figure 1b for Au825 (up) and Au800 (down). The Au NRs are single crystalline based on their TEM diffraction pattern (Figure 1c). The long axis of the NRs is parallel to the ⟨001⟩ crystallographic direction, and the surface is terminated by {730} facets (Figure 1d). The experimental extinction spectra (solid lines in Figure 1e) contain clear signatures of the so-called transverse and longitudinal plasmon resonances. The transverse (/longitudinal) plasmons have electric dipole perpendicular (/parallel) to the NR axis of symmetry (schemes in Figure 1e). In the same graph, the simulated extinction cross sections of transverse and longitudinal plasmons are depicted with dashed-dot and dashed lines, respectively. For this initial assessment of the optical properties we have simulated the geometry of the Au NRs as two spheres connected by a cylinder. Although the dispersion of Au NRs in PVA leads to unavoidable partial aggregation (see TEM from Figure S1), we cannot estimate the degree of aggregation from the extinction spectra due to their broad spectral shapes. According to Figure 1a and b, where the Au NRs were drop-cast from their water suspensions, aggregation of the Au NRs is also expected in PVA. Generally, the Au NRs are mostly expected to assemble side-by-side since the end-to-end configuration is energetically less favorable and requires specific surface ligands.^34^ Direct visualization with TEM of the Au NRs within the polymer is a difficult task, since the bulk insulating matrix attenuates the electron beam and deteriorates focusing.

**Figure 2:**
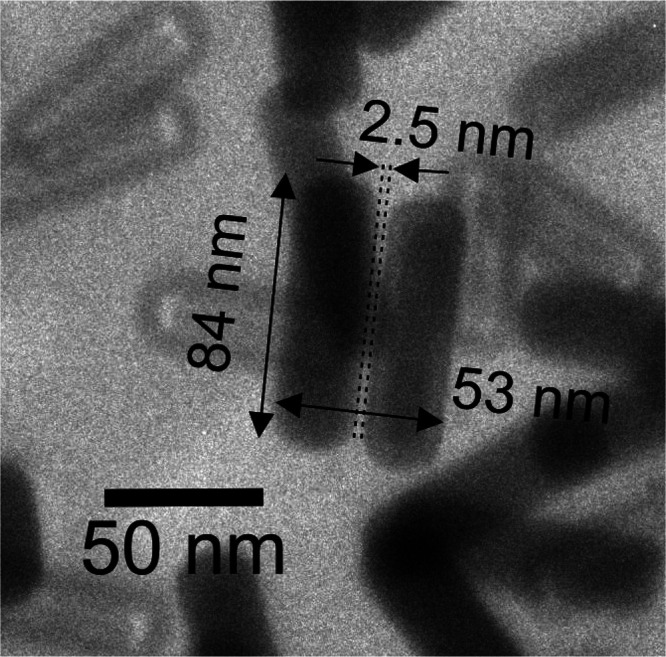
TEM image of a Au825 NR dimer in PVA showing side-by-side aggregation. The nanogap between the NRs is 2.5 nm wide.

Nevertheless, we repeatedly prepared thin slices of the Au NR–polymer nanocomposite until finding a part of the sample that is thin enough for TEM. Figure 2 shows a close image of a parallel dimer of Au825 NRs in PVA with a nanogap of 2.5 nm between them. Both NRs have the same length (84 nm) and diameter (25 nm), verifying that they both lie on the focusing plane. A TEM image at a lower magnification to show more examples of Au NR dimers in PVA can be found in the Supporting Information Figure S1.

### Brillouin Light Scattering Experiments

**Figure 3:**
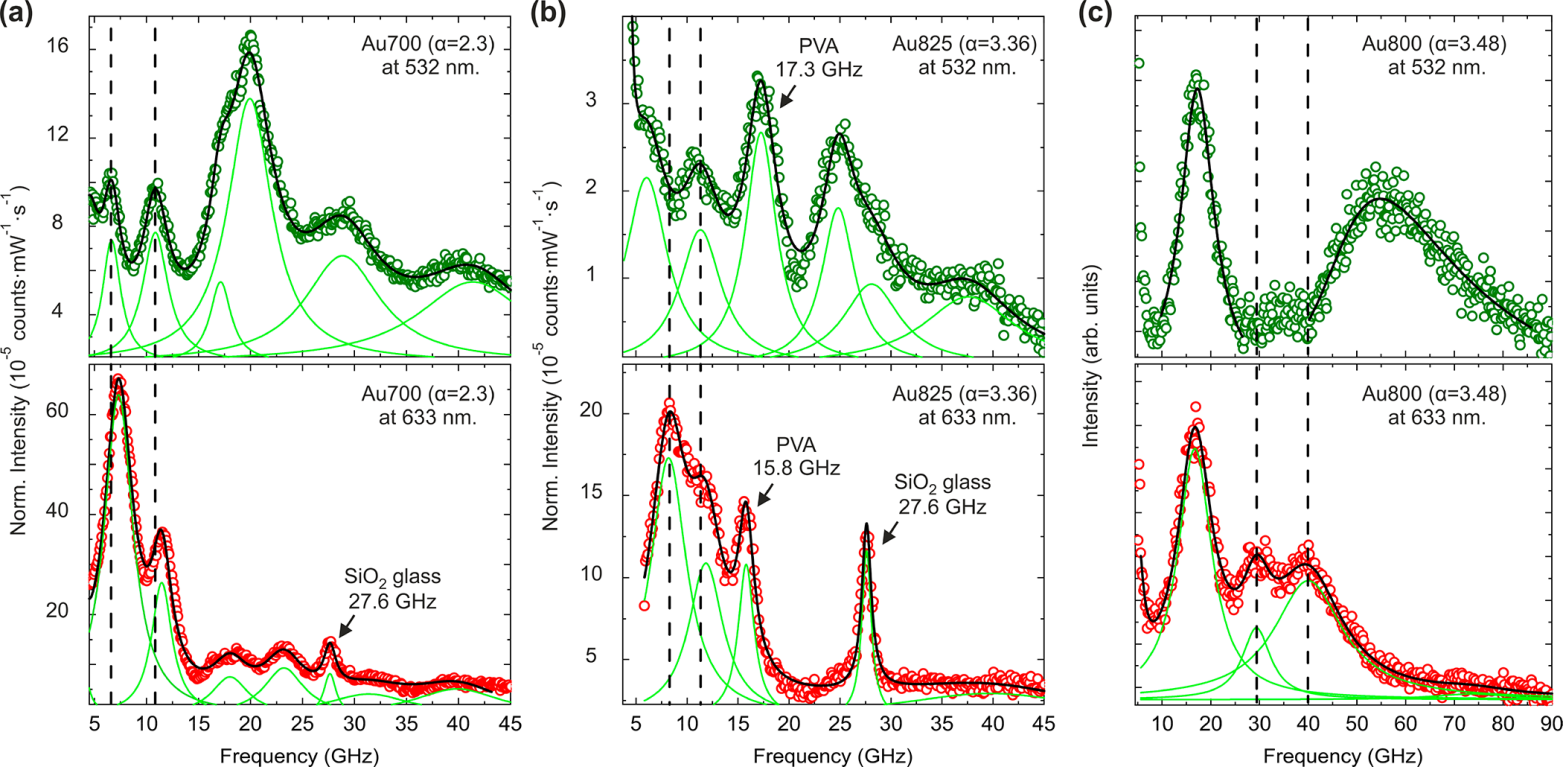
Wavelength-dependent inelastic light scattering spectra of Au NRs. (a–c) BLS spectra of Au700, Au825, and Au800 NRs with 532 nm (top) and 633 nm (bottom) CW light. The experimental data (points) are represented with a sum (solid black lines) of Lorentzian peak profiles (green lines). The measured BLS intensities have been normalized by the exposure time, the incident power, and the absorbance for Au700 and Au825, to quantify the plasmonic enhancement of the two lowest frequency modes.

Figure 3 shows the BLS spectra of Au700, Au825, and Au800 NRs with 532 nm (Figure 3a–c top) and 633 nm (Figure 3a–c bottom) laser light, respectively. The incident laser power is 0.5 mW for Au700 and Au825 (Figure 3a,b) and 1 mW for Au800 (Figure 3c). The experimental spectra (points) have been recorded in backscattering geometry and represented by a sum of Lorentzian peak profiles (Supporting Information S3). Inspection of Figure 3 leads to several interesting findings:

(1) In all cases, the BLS spectra strongly depend on the photon energy. Switching the wavelength of excitation light from 532 nm to 633 nm leads to the enhancement of the first two low-frequency peaks for Au700 and Au825 and to the emergence of new BLS peaks in the case of Au800. Note that for both wavelengths the BLS measurements are performed on the same sample spot. For monodisperse Au700 and Au825 samples, the BLS intensity is normalized with the incident laser power, the measured absorbance, and the exposure time to compare the scattering efficiency for the two laser sources. The enhancement factor of BLS for 633 nm compared to 532 nm is ∼10 (see Table S5). The enhancement of the low-frequency fraction of the spectra is complemented by a profoundly different pattern of the higher frequency above about 15 GHz (Figure 3a,b) and 20 GHz (Figure 3c) when recorded with 532 and 633 nm.

(2) Taking the fundamental extensional mode as reference, its frequency, , can be estimated by COMSOL analysis as well as analytically^19^ with the relationship
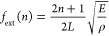
1where  is the length,  is the order of the mode,  is the density, and  is the Young’s modulus of the bulk isotropic gold. Relationship 1 refers to the so-called symmetric extensional modes that are BLS active, but there are additional BLS-inactive modes with a  dependence termed antisymmetric extensional modes.^35^ The fundamental extensional  for Au700, Au825, and Au800 is predicted to be at 12.3, 12.5, and 24.0 GHz. Experimentally, the nearest BLS peaks are located at ∼11.5, 11.5, and 17.3 GHz, respectively. The latter mode lies close to the PVA peak, and thus it was resolved with the depolarized (VH) BLS spectra. The experimentally observed frequencies are lower than the analytic model due to the anisotropic elastic properties of Au NRs, as well as the actual deviation from the cylindrical, flat-end shape of the analytic model. Noticeably, the analytic prediction for the breathing mode is, for instance, at 64 GHz for Au700 vs the experimental value at 62.7 GHz (Supporting Information Figure S4). Hence, all breathing modes fall outside the frequency ranges of Figure 3. For a single Au NR, the fundamental extensional mode is the BLS active mode of the lowest frequency. The appearance of spectral peaks below the fundamental extensional frequency can result from the vibrational and plasmonic coupling of aggregated nanostructures.^13−15^ The BLS peaks above the fundamental extensional frequency are contributed by either the trivial polymer matrix and glass substrate or various normal modes of Au NRs to be assigned by theoretical simulations.

(3) The plasmonic enhancement is pronounced for the coupling modes. Turning from green (532 nm) to red (633 nm) light, the peaks with frequency , stemming from coupled vibrational modes in NR aggregates (see next section), become stronger, whereas the peaks with  get comparatively much weaker. In the case of the high aspect ratio (α ≈ 3.4), the high-frequency peaks measured with 532 nm are hardly detected with 633 nm (Figure 3b,c). Compared to normal modes, coupled vibrational modes show a strong softening/frequency red-shift with increasing the incident laser power or equivalently the temperature.^13^ The sample heating due to laser light absorption from the Au NRs induces softening of the surrounding PVA matrix (Supporting Information Figure S5) that mediates coupling between Au NRs. Thus, laser-induced softening affects predominantly the coupling vibrational modes. Owing to this effect, we can identify that the lowest frequency mode, e.g., at ∼7 GHz for Au700, is a coupling mode. Note that such modes were not resolved in the literature from the BLS spectrum of close-packed Au NRs on a Si wafer recorded with 647 nm.^15^

(4) Normal Au NR modes with frequency  are clearly resolved in the spectrum of Au700 recorded with 532 nm (Figure 3a). Focusing on this spectrum with the richest structure and the most well-separated peaks, the quadrupolar mode, which is mainly determined by NR diameter, is BLS active^15^ and appears at ∼20 GHz. Experimentally, this BLS peak is better represented by a double Lorentzian (∼17 and 20 GHz), and it can contain a contribution from the bulk phonons in PVA or multiple Au NR modes. In fact, the spectral peak at about 17 GHz can be assigned to the effective medium longitudinal phonon in the PVA nanocomposite based on the BLS spectrum of Au700 NRs in PVA at 0.3 vol % (Supporting Information Figure S6).

The peaks at ∼30 and 40 GHz can be associated with higher order extensional modes. The Au825 NRs have a very similar length to Au700 but a smaller diameter, and, thus, the quadrupolar mode should be blue-shifted to ∼29 GHz. Experimentally, the spectrum of Au825 with 532 nm light (Figure 3b, top) displays a double Lorentzian with components at 25 and 30 GHz, respectively. This doublet is attributed to contributions of quadrupolar and extensional modes. Additionally, the BLS spectrum of Au825 also contains a peak at ∼37 GHz, close to the 40 GHz peak of Au700, conforming to a higher order extensional mode. For Au800 with the smallest length and diameter among the three Au NRs, its quadrupolar and higher order extensional modes aggregate at ∼50 GHz and above, giving a broad and antisymmetric BLS peak at 532 nm; see also Supporting Information Figure S7.

(5) For the low-frequency modes  that get enhanced with red light, we note that their frequencies are very close to the frequencies of NR bending modes as shown by COMSOL simulations (next section). The bending modes, which are BLS inactive for a single NR, become active as coupled transverse plasmons will break left–right symmetry. The transverse plasmons (∼530 nm) between neighboring NRs with side-by-side aggregation can hybridize and form bright coupled resonances at slightly longer wavelengths (∼600 nm). Thus, for 633 nm light, it is expected that the BLS intensity of coupled vibrational modes will be further enhanced. The existence of NR dimers is evident from the TEM images of Au825 in PVA (Figure 2).

### Optomechanical Calculations

Next, we proceed with the theoretical rationalization of these main experimental findings by presenting the possible (large number) mechanical modes of Au700 NRs in PVA, the plasmonic excitations, and the optomechanical coupling to identify the BLS active modes with strong plasmonic enhancement. In order for the calculated vibrational frequencies to match the observations, the model geometry must closely resemble the actual shape of Au700 NRs (Figure 1a). A close inspection of the Au NRs’ shape shows that their tips can be approximated as ellipsoids with an eccentricity of 0.866 (see Supporting Information). The same model geometry is used for the EM simulations. Finally, for the optomechanical calculations we evaluate the OM coupling rates arising out of the moving boundary and the photoelastic effects—the two main mechanisms describing optomechanical coupling and BLS—the sum of which is proportional to the single-photon optomechanical coupling rate^36,37^ (); see Methods.

**Figure 4:**
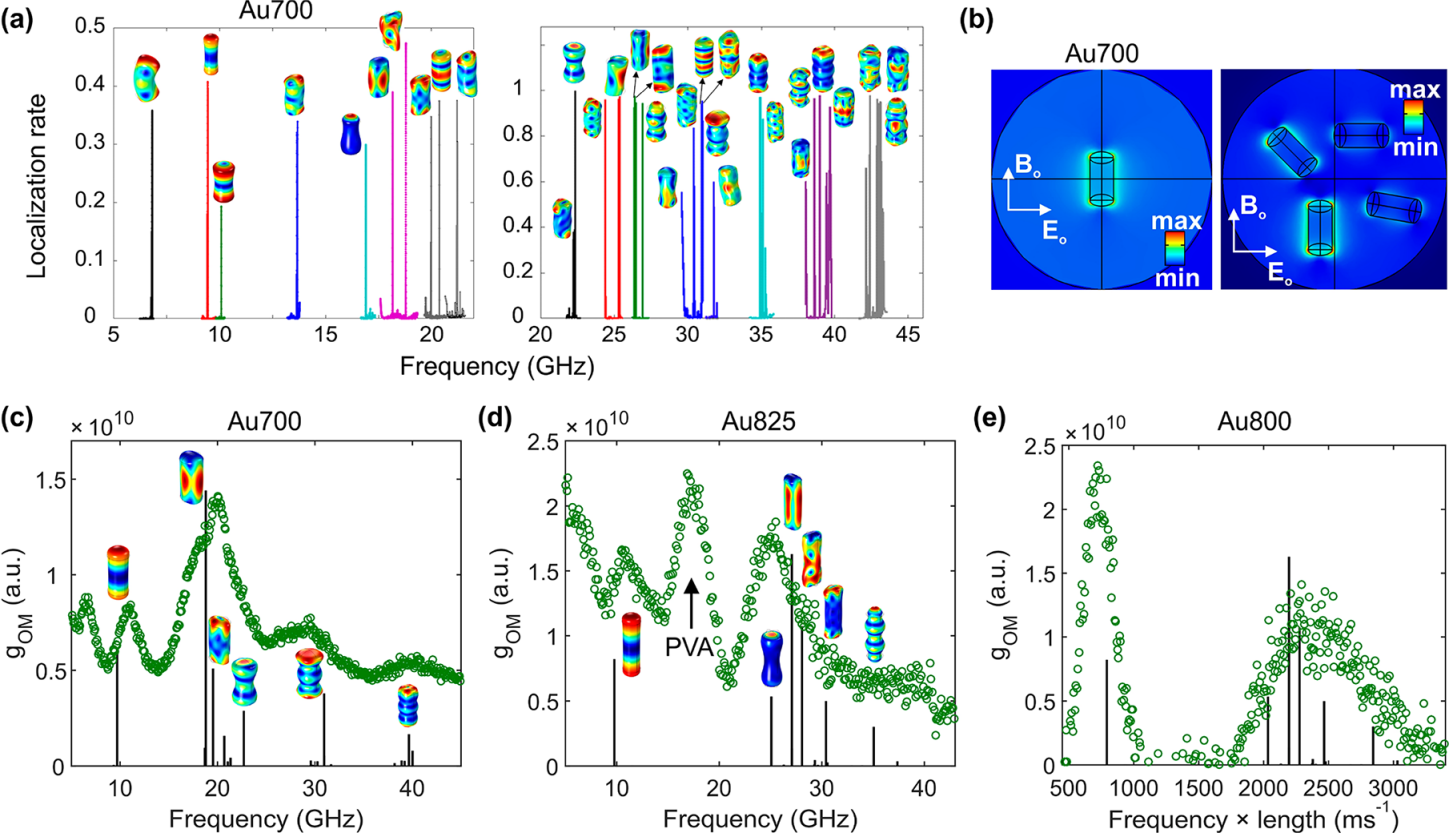
Eigenmodes, plasmons, and optomechanics of single Au NRs in PVA. (a) The localization rate as a function of the vibrational frequency revealing 32 eigenmodes of the Au700 monomer in PVA between 5 and 45 GHz. (b) The electric field distribution for a single Au700 NR with its long axis perpendicular to the incident electric field (left) and for a collection of Au700 NRs with different orientations (right). (c, d) The optomechanical coupling strengths (vertical bars) overlaid with the experimental BLS spectra (green dots) for comparison. The BLS active vibrational eigenmodes of Au700 and Au825 are shown as insets in (c) and (d), respectively. The strong peak at about 17 GHz in (b) is the effective medium longitudinal phonon in the polymer (PVA) matrix. (e) The optomechanical coupling spectrum for Au825 (vertical bars) overlaid with the BLS spectrum of the Au800 NRs (green dots) as a function of the frequency multiplied by the respective NR length. Au800 and Au825 NRs have very similar aspect ratios (α ≈ 3.4).

Figure 4 presents the eigenmodes of Au700 NRs in PVA up to 45 GHz, the calculated plasmonic near-fields for various NR orientations, and the optomechanical calculations that identify which of all possible eigenmodes are BLS active. Based on the localization rate (Figure 4a), there are 32 eigenmodes of various characters with their shape shown in the insets. In the range 5 to 21 GHz (Figure 4a left) the fundamental bending mode (*n* = 0) is at 6.8 GHz, while higher order bending modes are visible at 13.6 GHz (*n* = 1) and 21 GHz (*n* = 2). The fundamental extensional mode (*n* = 0) is at ∼9.5 GHz, and the first antisymmetric extensional (*n* = 1) at 16.9 GHz. The torsional modes, which are BLS inactive, appear at 10 GHz (*n* = 0) and ∼20 GHz (*n* = 1). The first quadrupolar mode appears at ∼18 GHz, and additional quadrupolar-like modes emerge at 18.7 and 19.9 GHz. The density of eigenmodes is drastically increasing as we move to higher frequencies (Figure 4a right). The same normal modes are found at similar frequencies for a Au700 monomer in air, and their shapes are shown in Figure S8.

Turning to the numerical calculations of the plasmonic near-fields, the left panel of Figure 4b shows the electric field norm for an incident plane wave , with propagation direction  and polarization  normal to the NR axis (*z*-axis). This geometry leads to almost resonant excitation of transverse plasmons, and it represents the dominant contribution to the total BLS signal. As shown in the right panel of Figure 4b, the light is strongly focused on the NRs that are predominantly oriented normal to , while the other orientations remain dark. Therefore, for the green laser at  corresponding to the resonance wavelength of the transverse plasmon of a single NR (monomer), the strongest OM coupling will be mainly generated from the ensembles of NRs aligned perpendicularly to the incoming field  (excitation of transverse plasmon). The NRs with different orientations will contribute much less to the overall signal.

The next step is to find the optomechanical coupling for each eigenmode. Figure 4c shows a comparison between the calculated, mode-resolved  of Au700 NRs with a 532 nm wavelength of light and the experimental BLS spectrum of Au700 measured with 532 nm light. Noticeably, only seven eigenmodes produce strong optomechanical coupling up to 50 GHz, and all the rest are BLS inactive. The optomechanical calculations predict the emergence of BLS peaks at approximately 10, 20, 30, and 40 GHz, in close agreement with the experiment. They also verify the initial interpretation of the experimental Au700 spectra with 532 nm light (Figure 3a top and relevant discussion) as fundamentally extensional at approximately 10 GHz, primarily quadrupolar at ∼20 GHz, and primarily higher order extensional at 30 and 40 GHz. As noted before, the bending mode of the Au700 monomer is BLS inactive and cannot explain the experimental peak at ∼7 GHz. To understand how the bending mode becomes BLS active, we need to simulate the vibrations, plasmons, and optomechanical coupling of NR dimers.

The same theoretical procedure can be used to interpret the BLS spectra of Au825 at 532 nm (Figure 3b top). Au825 has 18 eigenmodes between 5 and 40 GHz (Figure S9), out of which only six produce strong optomechanical coupling. The optomechanical calculations (Figure 4d) yield the first extensional mode at 9.8 GHz, the second extensional mode at 25.1 GHz, two strong quadrupolar-like modes close to 27 GHz, and a higher order extensional mode at 35 GHz. These frequencies are in good agreement with the experimental BLS spectrum of Au825, which can be represented with four Lorentzian peaks at 11.3, 24.8, 28.0, and 37.6 GHz (Table S3). As the Au825 has a similar aspect ratio to Au800 (α ≈ 3.4), their optomechanical and BLS spectra overlap when plotted as a function of frequency multiplied by the respective NR length (Figure 4e).

**Figure 5:**
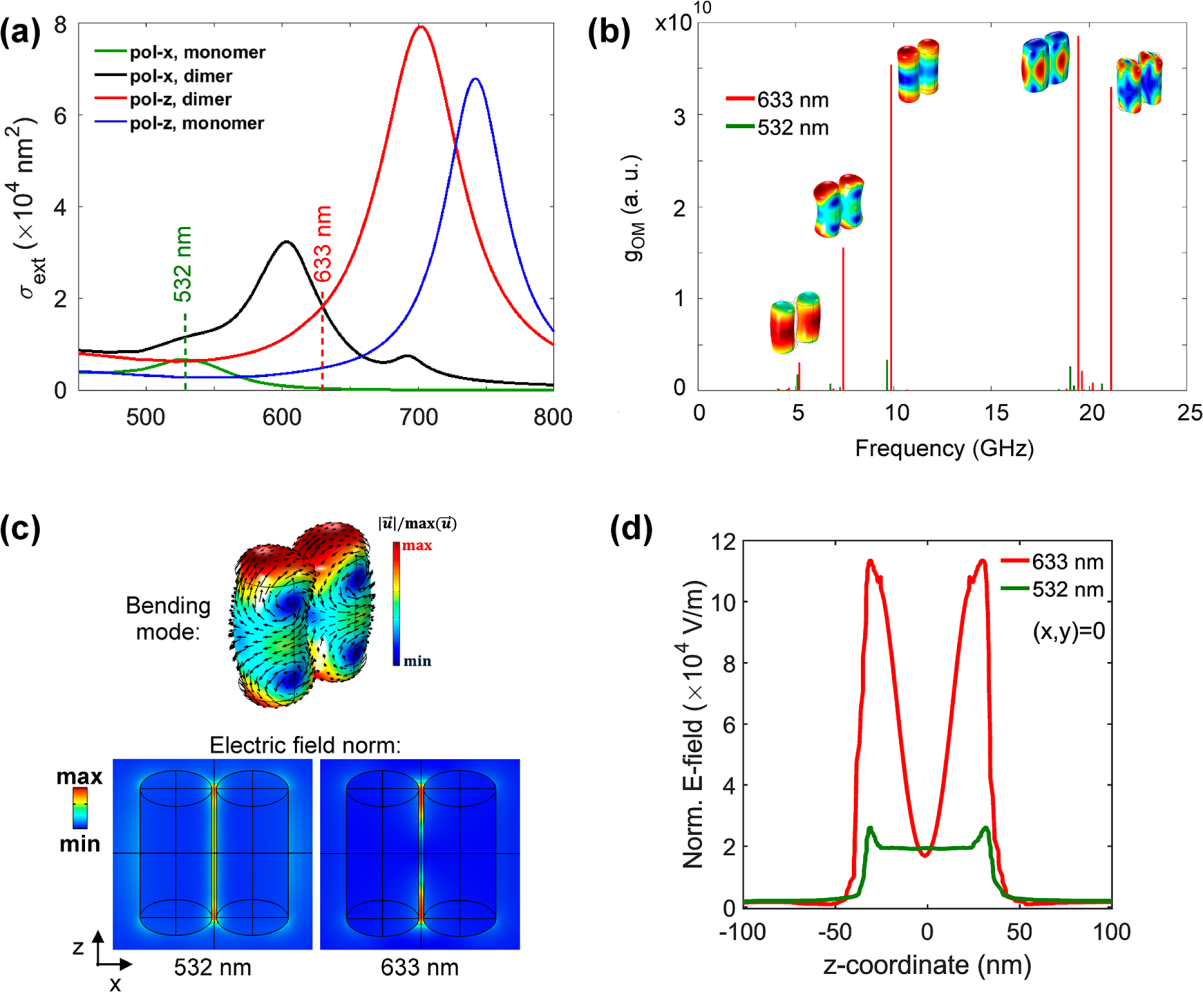
Plasmonic coupling and optomechanical calculations of the Au700 dimer in PVA. (a) The extinction cross-section as a function of wavelength for the Au700 monomer with *x*-polarization (green) and *z*-polarization (blue) and the Au700 dimer with polarization along the *x*-axis (black) and the *z*-axis (red). All these calculations employ the ellipsoid-capped NR geometry of the optomechanical model. The vertical dashed lines denote the wavelength of the laser excitation. (b) The optomechanical spectrum up to 25 GHz for the Au700 dimer averaged over the *x-* and *z*-polarizations. (c) The displacement field of the dimer bending mode (top) and the plasmonic near-fields for the two laser wavelengths (bottom) with *x*-polarized incident light. (d) The norm of the electric field along the *z*-coordinate, with  for the two laser wavelengths with *x*-polarized incident light.

Figure 5a shows the extinction spectra for the Au700 monomer with *x*-polarization (green, the incident electric field is vertical to the NR) and *z*-polarization (blue, the incident electric field is parallel to the NR). The same plot shows the extinction spectra for the Au700 dimer with *x*- (black) and *z*-polarization (red, the incident electric field is parallel to the NR). The nanogap between the two Au NRs is 2.5 nm wide, according to the TEM image in Figure 2. As expected, the bright coupled plasmons are red-shifted for the transverse and blue-shifted for the longitudinal plasmon resonances compared to the individual Au700 NRs. The red laser at 633 nm is off-resonance with respect to both transverse and longitudinal plasmons, and the two coupled plasmons have similar extinction at this wavelength. Thus, the OM coupling for the dimer and for both lasers was averaged on both polarization sets (along  and ) by taking into account the random NR orientations with respect to .

Evidently, the formation of dimers is expected to enhance the absorption of 633 nm light (vertical line in Figure 5a) and, thereby, the BLS signal. Indeed, the optomechanical coupling is enhanced for all modes up to about 20 GHz (Figure 5b). The direct comparison between the calculated  and the experimental BLS spectra would require additional considerations, such as to modulate the spectra by the thermal occupation factor and to know the relative abundance and BLS contribution of monomers and dimers. Yet, the bending modes are BLS inactive for the Au NR monomers, and thus we can use this mode to understand the origin of plasmonic (wavelength-dependent) enhancement for the Au NR dimers. In this context, it should be noted that the optomechanical coupling strengths for radiation at both 633 nm (Figure S10) and 532 nm are quite similar. Thus, aggregates of Au NRs with modified plasmonic near-fields are necessary to explain the BLS enhancement.

Theoretically, the  for the dimer bending mode at ∼7 GHz shows 44-fold enhancement excited with a laser at 633 nm compared to that at 532 nm (second depicted mode in Figure 5b). The corresponding experimentally observed enhancement factor (Figure 3a) is smaller (11.7), probably reflecting the dimers population in the sample. The monomer modes are still discernible in the latter spectrum recorded at 633 nm (above 15 GHz in Figure 3a, bottom) due to the contribution of the longitudinal plasmon (red arrow in Figure 1e). The strong enhancement at the frequency of ∼12 GHz is likely due to the corresponding interaction mode at very similar frequency (Figures 4c and 5b).

For a qualitative understanding of the plasmonically enhanced OM coupling and BLS, we note that the displacement field of the symmetric coupled bending mode is maximized close to the NR dimer tips (Figure 5c top) and so are the plasmons at 532 and 633 nm (Figure 5c bottom). The wavelength dependence of BLS can thus result from the reshaping of the plasmonic near-fields when moving from 532 nm to 633 nm light. To further illustrate the wavelength-dependent change of the plasmonic near-fields, Figure 5d shows the norm of the total electric field () for -polarized incident light. The  is plotted as a function of  and for  (the middle between the two NRs).

**Figure 6:**
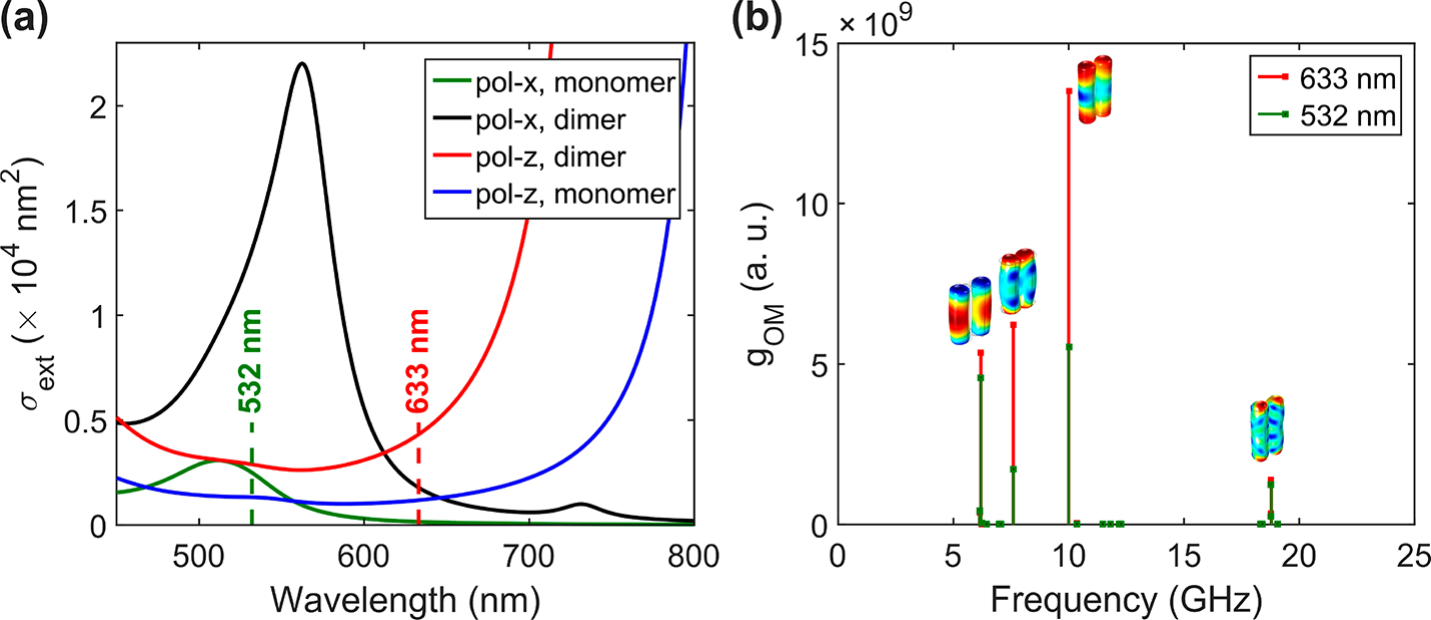
Plasmonic coupling and optomechanical calculations of the Au825 dimer in PVA. (a) The extinction cross section as a function of wavelength for the Au825 monomer with *x*-polarization (green) and *z*-polarization (blue), and the Au825 dimer with polarization along the *x*-axis (black) and the *z*-axis (red). (b) The optomechanical spectrum up to 25 GHz for the Au825 dimer averaged over the *x-* and *z*-polarizations.

The theoretical procedure followed for Au700 dimers yields similar results in the case of the Au825 system, as well. Figure 6 shows the calculations of plasmonic and optomechanical coupling in a Au825 dimer as that observed in the TEM image of Figure 2. The coupled transverse and longitudinal plasmons enhance the absorption at 633 nm (Figure 6a), compared to the transverse plasmon of individual Au825 NRs (see also Figure S11). Based on the optomechanical calculations (Figure 6b), the lowest frequency BLS peak of Au825 at ∼8 GHz (Figure 3b, bottom) is assigned to coupled bending modes, which get enhanced with off-resonant light at 633 nm due to plasmonic coupling. However, unlike Au700, normal modes of Au825 monomers are not discernible (Figure 3b, bottom) due to off-resonance conditions between the longitudinal plasmons (∼825 nm) and the 633 nm light (Figure 1e, red arrow).

Finally, in the case of Au800, the lowest frequency bending mode (below 10 GHz) cannot be resolved due to the strong Rayleigh scattering (Figure 3c), while the new peaks observed with radiation at 633 nm are attributed to higher order coupling modes. The plasmonic coupling, leading to the appearance of new BLS peaks with off-resonant light at 633 nm, is evident from the absorption spectrum of Au800 in PVA (Figure S12). The transverse plasmon resonance is asymmetrically extended toward longer wavelengths due to the presence of coupled transverse plasmons.

## Conclusions

Plasmonic coupling in aggregated nanostructures can modify the BLS selection rules.^13−15,17^ In some cases, the spectroscopic signatures of coupled modes were either detectable or completely absent depending on the wavelength of light, while in other cases, the breakage of selection rules was only shown for the nanoparticles’ normal modes. Here, we examined Au NR monomers and dimers dispersed in PVA both in theory and experimentally, which give plasmon-enhanced BLS at various laser wavelengths. The experimental BLS spectra are enhanced with off-resonance light (with respect to the transverse plasmons) due to plasmonic coupling between Au NRs. These BLS results were interpreted using OM calculations.

The OM coupling is a measure of the spatial overlap between the electric field and the mechanical displacement; see for instance, eq 6 (Methods) for the moving interface effect. Alteration of the BLS selection rules and modification of the plasmonic enhancement requires that the coupled plasmon near-fields lower the overall symmetry compared to plane EM waves. In this context, additional types of aggregates,^38^ e.g., L-shaped end-to-end configurations, or rod-like structures with prescribed symmetries and distances are promising nanostructures for the exploration of the optomechanical interactions using micro-BLS with good spatial resolution.

Here, we have limited our analysis to the parallel dimers observed in PVA with TEM (Figure 2 and Supporting Information). For the coupled bending mode of parallel dimers, the displacement is maximized close to the NR tips (Figure 5c). With the aid of theoretical calculations, we have visualized the EM fields at various photon energies and revealed energy-dependent confinement of the coupled plasmons close to the tips of NR dimers. The near-fields of the coupled transverse plasmons show stronger confinement toward the NR tips for 633 nm compared to 532 nm light (Figure 5d). This wavelength-dependent shape of plasmonic coupling can explain the drastically different BLS spectra measured for 532 nm versus 633 nm wavelength of light (Figure 3).

Taking into account that the OM coupling is roughly proportional to the electric field squared , the characteristic size of these hot spots is on the order of 15 nm for a 633 nm wavelength of light. This length is a rough estimation from the fwhm of the  distribution (red line in Figure 5c). In the case of 532 nm wavelength of light, the  distribution is more delocalized (green line in Figure 5c), the OM coupling samples a larger part of the coupled bending displacements, and the BLS signal is weaker.

In summary, BLS can be used to reveal plasmonic coupling and OM hot-spots in plasmonic nanoparticle–polymer nanocomposites. Plasmonics offer promising ways for developing ultrasensitive spectroscopic techniques, especially for single-molecule detection with surface-enhanced Raman scattering (SERS). The tremendous enhancement of SERS is now associated with highly localized EM fields described as picocavities and atomic-scale lightning rods.^39^ The BLS signal of confined acoustic modes can also be enhanced through plasmons, but the observed enhancement factors are generally smaller by orders of magnitude than that of SERS. Contrary to SERS from molecules, the BLS signal can originate from the entire volume and surface of nanostructures, and it depends on the overlap and symmetries of the plasmonic near-field and the vibrational displacement field. Here, we have shown that plasmonic coupling between Au NRs can focus the EM fields on specific parts of the displacement field and give a 10-fold BLS enhancement.

From the experimental point of view, an exciting prospect is to employ BLS microscopy^40^ to spatially resolve hot-spots and plasmonic couplings in nanocomposites. On the side of theory, the presented computational methods can be used to describe BLS from plasmonic nanostructures of arbitrary shape, beyond nanospheres,^33^ which is a tool that was missing, as noted in recent BLS studies.^15^ The use of BLS as an optomechanical probe of plasmons can be useful for characterizing acousto-plasmonic devices with Au NR–polymer nanocomposites. We note that PVA, the matrix chosen in this study, is inert and does not have chemical interactions with Au NRs, as our first focus is on Au NRs themselves. By selecting a different inert matrix (PVP), we have observed the same modification of BLS spectra in response to the laser wavelength (Supporting Information Figure S13). Given the broad range of polymers that we can choose, we envision various opportunities. For instance, the soft polymer matrix can host propagating acoustic waves, which can then excite the Au NR assemblies, modulate the nanogaps between Au NRs, and get detected by means of optomechanics and BLS. Another possibility is to employ stimuli-responsive polymers. For instance, the PVA can be subjected to laser-induced heating and softening while simultaneously probing the temperature via BLS (Supporting Information S8). Such methods can be particularly interesting for thermoplasmonics^28^ and nanocomposites with liquid crystal elastomers.^30,41^

## Methods

### Fabrication

To synthesize the Au NR-PVA nanocomposites, we used standard and modified seed solution recipes^42,43^ that provide single-crystalline NRs with well-defined surface facets.^44^ Briefly, the Au seeds were prepared by reduction of HAuCl_4_ with NaBH_4_ in cetyltrimethylammonium bromide (CTAB). The NR growth occurred after mixing the seeds with an aqueous solution of HAuCl_4_ containing surfactants, shape controlling agent, and reducing agent (see Supporting Information S1). Finally, the Au NRs were dispersed in the water-soluble PVA and drop cast on 1 mm thick microscope silica glass slides. The films had a dense red coffee ring with a semitransparent interior. The optical properties of the samples were characterized with a UV/vis spectrophotometer and locally with a white-light optical microscope fiber-coupled to an AvaSpec-HERO spectrometer. The nanoscale morphology and crystalline structure of the NRs, prior to their dispersion in PVA, were captured by TEM (Figure 1a,b), electron diffraction (Figure 1c), and high-resolution TEM (HR-TEM) (Figure 1d). The TEM images and diffraction in Figure 1 were obtained for Au NRs drop cast from the aqueous suspension, for the purpose to obtain size, shape, and crystallinity. To visualize the dispersion of Au NRs in PVA, we have examined thin slices of the final, nanocomposite samples with TEM. Based on volume fraction, the average distance between the neighboring NRs is estimated as ∼250 nm. In spite of the homogeneous dispersion, NR aggregation in PVA was unavoidable (Figure 2).

### Brillouin Light Spectroscopy

The BLS measurements were conducted in the backscattering geometry (Figure S2) with a six-pass tandem Fabry–Pérot interferometer (JRS Optical Instruments) with continuous wave (CW) lasers operating at λ = 532 nm and λ = 633 nm. The first laser is nearly resonant with the transverse plasmons (≈525 nm, Figure 1c) for all three NRs, while the second laser is off-resonant but within the expected energy range of bright coupled transverse plasmons. The momentum of bulk phonons probed with BLS is , where  is the refractive index of the polymer matrix. The maximum incident power to avoid irreversible changes was 3 mW. The incident (V)–scattered (V or H) light was s–s (VV) or s–p (VH) polarized with V (H) denoting vertical (horizontal) polarization with regard to the scattering plane. In the VH case, the signal from the bulk polymer was suppressed. Additionally, the pure polymer without NRs has been studied with BLS in backscattering and 90A transmission geometry (). The thermally induced softening of the PVA matrix has been studied on a Linkam device (Figure S3).

### Optomechanical Calculations

Due to the single-crystalline nature of the NRs (see Figure 1c), the mechanical properties of gold have been described with the anisotropic elasticity matrix^45^ and density . PVA has been described by its Young’s modulus , density , and Poisson’s ratio . For the optical properties of PVA, we have used the wavelength-dependent refractive index measured by Schnepf et al.^46^ For Au, we have employed the Drude–Lorentz model within which the dielectric constant is given by^4^
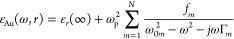
2

Here,  is the bulk plasma frequency,  is the interband transition frequency associated with the  oscillator,  is the weight of the latter, and  is its relaxation rate. The values of these parameters have been set by fitting eq 2 to experimental data.^4,47^ The frequency-domain electromagnetic and mechanical eigenfrequency simulations were carried out with the finite element method (FEM) using the commercially available RF and solid-mechanics modules of COMSOL Multiphysics, respectively. In particular, to get the mechanical eigenmodes of the single or dimer NRs in a PVA matrix, we simulated the energy localization rate defined as the ratio of elastic energy within the NR and in both the latter and the surrounding PVA matrix, such as
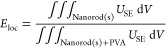
3where  is the strain energy density, which reads for an elastic anisotropic material as follows:
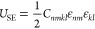
4

The  are the components of the fourth-order stiffness tensor of the material and  (or ) are the strain tensor components. Regarding the electromagnetic scattering problem, we have solved for the scattered field such that the background field in the absence of the NR was simply given by the incoming field () within PVA:
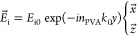
5

Here,  are Cartesian coordinates with the -axis parallel to the NR axis of symmetry,  is the PVA refractive index, and  is the wavenumber in vacuum. It should be noted that in our problem we have considered two possible polarizations for the incoming light, namely,  was set perpendicular or parallel to the NR axis. In both cases, the incident field propagates along the *y*-axis. The choice of these two polarizations sets will be made explicit a little further.

The OM coupling coefficients could be described with the perturbation theory of Johnson et al.^48^ and with the dissipative perturbation theory,^49,50^ which was adapted for plasmonic systems. Nevertheless, in such theoretical frameworks, both the electric and the mechanical fields correspond to eigenmodes, for which one aims at determining the degree of overlap. In order to be able to employ the scattered field (instead of the plasmon quasi-normal eigenmode^51^) and the displacement of the mechanical eigenmode of interest, we followed a slightly different framework. We evaluated the OM coupling rates arising out of the moving boundary and the photoelastic effects, with two overlap integrals involving the superposition of the electromagnetic and mechanical fields and took their sum as being proportional to the single-photon optomechanical coupling rate^36,37^, that is, .

The surface and volume along which the integrals were evaluated correspond to those of the NR considered as a cavity. A more detailed discussion on this selection is provided in the Supporting Information, S9. The integrands  and  refer to the surface density OM force based on the moving boundary effect and to the volume density of the OM force arising from the photoelastic effect, respectively. Their expressions are given by^36^
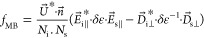
6
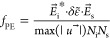
7where  is the normalized displacement field (*u⃗* being the displacement field), , ,  is the PVA dielectric constant,  is the outside normal to the boundary;  and  are material interface tangential electric and normal displacement fields for the incident (*j* = i) or scattered (*j* = s) field. In the relationship eq 7,  is the amount by which the NR permittivity is modified due to acoustic strain. The physical mechanism behind this parameter and its mathematical expression has already been detailed elsewhere.^4,52^ At last,  () is a normalization factor given by . Let us mention that we dropped such parameters in the denominator. The reason for that is as follows: in order to model different possible orientations of a NR within the matrix with respect to the incoming field, we consider for the latter two possible sets of light polarizations. Those are the polarizations states mentioned above, i.e., along  (i) or parallel to  (ii). At resonance, the polarization (i) can excite the transverse plasmon, whereas (ii) yields the longitudinal plasmon. The quantity  depends on each polarization set, in which case the computed integrals  will be weighted differently depending on the polarization. For that matter, and in order to avoid mistakenly weighting of the total OM force, we simply dropped out  and computed  in arbitrary units.

The electric field distribution in the right panel of Figure 4b also makes clear why the denominators of relationships 6 and 7 need to be omitted in the calculations. The 532 nm light will be more strongly focused on the Au NRs whose long axis lies vertical to the polarization of light, which will contribute most of the BLS signal. Yet, the normalization procedure will lead to an artificial equalization of the OM couplings from all Au NRs, as the Au NRs with different orientations will be divided by a smaller amount of stored electromagnetic energy. The same argument applies when comparing monomers with dimers or different wavelengths of light. Instead, we perform all calculations with the same amount of incident laser power and simply drop out normalization factors in the expression of .

## Supporting Information Available

Sample synthesis and characterization, TEM images of Au NRs in PVA, additional information on the BLS technique, momentum-resolved and temperature-dependent BLS spectra of PVA, peak parameters used to represent the BLS spectra, plasmonic enhancement factors of BLS, BLS peak of the breathing mode for Au700 in PVA, BLS measurements of laser-induced softening of coupled vibrational modes, BLS of Au700 in PVA at lower concentration, polarized (VV) and depolarized (VH) BLS spectra of Au800 with 532 nm light, normal modes of Au700 monomer in air, normal modes of Au825 monomer in PVA, optomechanical calculations with 633 nm radiation for single Au700 and Au825 NRs, theoretical extinction spectra of Au825 between 700 and 900 nm, absorption spectrum of Au800 in PVA showing experimental evidence of plasmonic coupling, observation of plasmonic BLS enhancement for Au800 NRs in PVP, temperature effect in Au800–PVA nanocomposites, additional details on the optomechanical model (PDF)
