# Supplementary material for: Optomechanical Hot-Spots in Metallic Nanorod–Polymer Nanocomposites
Source: ACS Nano. 2022 Dec 7;16(12):20419–29. doi: 10.1021/acsnano.2c06673 (PMC9798866; doi:10.1021/acsnano.2c06673)
Supplement: Supplementary file 1 — nn2c06673_si_001.pdf [file nn2c06673_si_001.pdf]

Supporting Information:
Optomechanical hot-spots in metallic nanorod-polymer nanocomposites

Thomas Vasileiadis[1]*, Adnane Noual[2], Yuchen Wang[3], Bartlomiej Graczykowski[1,4], Bahram Djafari-Rouhani[5], Shu Yang[3], George Fytas[4]* .

1. Faculty of Physics, Adam Mickiewicz University, 61-614 Poznan, Poland.
2. LPMR, Département de Physique, Faculté des Sciences, Université Mohammed Premier, Oujda, 60000, Morocco.
3. Department of Materials Science and Engineering, University of Pennsylvania, 3231 Walnut Street, Philadelphia, PA 19104, United States.
4. Max Planck Institute for Polymer Research, 55128 Mainz, Germany.
5. Département de Physique, Institut d'Electronique de Microélectonique et de Nanotechnologie, UMR CNRS 8520, Université de Lille, Villeneuve d'Ascq, 59655, France.

S1. Fabrication of gold nanorod – polymer nanocomposites.

Materials: Cetyltrimethylammonium bromide (CTAB, ≥98%, $C_{19}H_{42}BrN$), gold(III) chloride trihydrate ($HAuCl_4{\cdot}3H_2O$), silver nitrate (≥99%), L-ascorbic acid (BioUltra, ≥99.5%), Poly(vinylpyrrolidone) (PVP, M_w = 29,000 g/mol), and poly(vinyl alcohol) (PVA, M_w = 13,000 - 23,000 g/mol, 87-89% hydrolyzed) were purchased from Sigma-Aldrich. Sodium Oleate (NaOL) was purchased from TCI America. Hydrochloride acid (HCl) and nitric acid (HNO_3) were purchased from Fisher Scientific. Sodium borohydride ($NaBH_4$) was purchased from Fluka. Ultrapure water produced by a Milli-Q Integral 5 system was used in all experiments. All glassware and stir bars were cleaned with aqua regia (3:1 v/v HCl (37%): HNO_3 (65%) solutions), followed by extensive rinsing with deionized (DI) water, and dried before use.

Synthesis of Gold nanorods (Au NRs): The seed solution for Au NR growth was prepared as follows: 0.25 mL of 10 mM $HAuCl_4{\cdot}3H_2O$ was mixed with 10 mL of 0.1 M CTAB solution in a 20 mL scintillation vial. 0.6 mL of fresh 0.01 M $NaBH_4$ was then injected under vigorous stirring (1200 rpm). The solution color changed from yellow to brownish yellow and the stirring was stopped after 2 min. The seed solution was aged at room temperature for 30 min before further use.

We have prepared three different samples of Au NRs in PVA that are named according to the wavelength of light that excites their longitudinal surface plasmon resonance (LSPR). They are: Au700 with length 82.1 ± 4.2 nm and diameter 35.7 ± 2.3 nm, Au825 with length 81.1 ± 5.7 nm and diameter 24.1 ± 1.3 nm, and Au800 with length 42.5 ± 4.5 nm and diameter 12.2 ± 2.9 nm. The code of each sample denotes the wavelength of the longitudinal plasmon resonance in nanometers: Au700, Au800 and Au825 have longitudinal plasmon resonances at approximately 700, 800 and 825 nm, respectively.

To prepare the growth solution for Au825 NRs and Au700 NRs: 1.75 g CTAB and 0.3085 g of NaOL were dissolved in 115.4 mL of warm water (~50 °C) in a 250 mL flask. The solution was allowed to cool down to 30 °C and 2.4 mL 10 mM $AgNO_3$ aqueous solution was added. The mixture was kept undisturbed at 30 °C for 15 min, after which 6.25 mL 10 mM $HAuCl_4 \cdot 3H_2O$ was added. The solution became colorless after 90 min of stirring (700 rpm). Then 0.525 mL HCl (37 wt%) was added. After another 15 min of slow stirring at 400 rpm, 0.2 ml 0.1 M ascorbic acid was added and the solution was vigorously stirred for 30 s. Finally, 0.2 mL seed solution was injected into the growth solution. The resultant mixture was stirred for 30 s and left undisturbed at 30°C for 12 h for NR growth. For Au700 NRs, 1.2 mL 10 mM $AgNO_3$ aqueous solution (or 2.4 mL for Au825) was added while keeping other procedures the same. The final products were isolated by centrifugation at 6,000 rpm for 20 min followed by removal of the supernatant.

To prepare the growth solution for Au800 NRs: 10 mL of 0.01 m $HAuCl_4 \cdot 3H_2O$ and 1.5 mL of 0.01 M $AgNO_3$ were added to 237.5 mL of 0.1 M CTAB in a 250 mL flask. The solution was slowly stirred for 15 min followed by adding 1.6 mL of 0.1 M ascorbic acid. Finally, 1.8 mL seed solution was added into the mixture. The solution was gently mixed and left at 30 °C for at least 3 h. The final products were isolated by centrifugation at 9000 rpm for 20 min followed by removal of the supernatant. The AuNR/PVA and AuNR/PVP films were obtained by drop casting 1 wt% PVA aqueous solution containing 1 vol% AuNRs on the precleaned glass slides. TEM images shown in **Figure 1** in the main article were taken on a JEOL JEM-F200 at 200 kV.

S2. TEM image of Au825/PVA nanocomposites.

For characterization of transmission electron microscopy (TEM) images of Au NRs dispersed inside the PVA matrix, we have removed several pieces of the AuNR – polymer nanocomposite using a scalpel and a tweezer and placed them between two commercially available copper TEM grids. These samples were examined by JEOL ARM 200F at an accelerating voltage of 200 kV until we locate an area that is thin enough for TEM imaging. A low magnification image of Au825 NR is shown in **Figure S1a**. From this image we could identify several cases of side-by-side aggregated NR dimers (**Figure S1b**). An image of a Au825 NR dimer at higher magnification is shown in **Figure 2** of the main article.

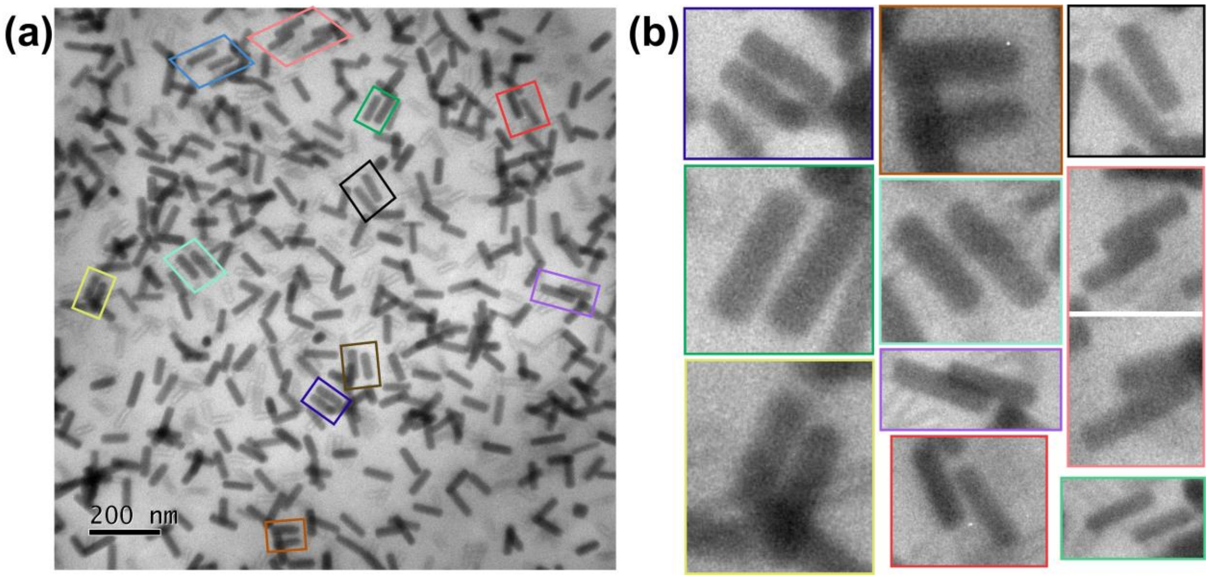

Figure S1: (a) TEM images of Au825 dispersed in PVA at a low magnification. **(b)** Examples of NR dimers with side-by-side aggregation.

S3. Brillouin Light Scattering (BLS) spectroscopy.

The Brillouin light scattering (BLS) spectroscopic measurements were carried out in the backscattering geometry shown in **Figure S2** using the glass slides with the AuNRs – PVA nanocomposites on their surface. For the BLS experiments we have used single mode, continuous wave (CW) lasers with either 532 nm (Spectra-Physics, Excelsior 300) or 633nm (Newport HeNe) wavelength of light. The maximum incident power to avoid irreversible changes was estimated to be 3 mW. At higher power the laser would gradually induce mass-transfer and formation of a visible crater at the irradiation spot. The light was focused on the sample with a ×50 microscope objective. The use of a ×10 microscope objective yielded similar results.

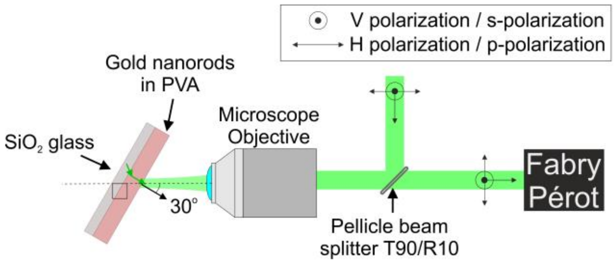

Figure S2: Scheme of Brillouin light scattering (BLS) in backscattering geometry and illustration of the polarization geometries.

Using two linear polarizers and two lambda half waveplates, we could select the incident – scattered light polarizations. The polarized BLS spectra were measured with s-s (VV) or p-p (HH) polarization, which are identical for the system investigated here. The depolarized BLS spectra were measured with s-p (VH) or p-s (HV) polarization. When the effective medium dominated the BLS spectra, we measured depolarized BLS to suppress its contribution and to reveal the AuNR vibrations – see for instance **Figure 3c** in the main article.

The spectrum of the inelastically scattered light has been recorded with a Fabry–Pérot interferometer (JRS Scientific Instruments). The entrance pinhole for the spectrometer was 200 μm. The mirror spacing was 1.5 mm. The scanning amplitude was 490 nm (/600 nm) for 532 nm (/633 nm) wavelength of light. The normal modes of the Au NRs are confined acoustic vibrations for which momentum-transfer during BLS is not relevant. The momentum-resolved BLS measurements that were used to study the effective medium properties are presented in the following section.

Momentum-resolved and temperature-dependent BLS of PVA:

In addition to the BLS studies of the normal modes of Au NRs in PVA, we have performed momentum-resolved and temperature-dependent BLS studies of pure PVA. **Figure S3** describes the temperature-dependent BLS measurements of pure PVA. The measurements were carried out in backscattering and 90A transmission geometry (**Figure S3a**). In the 90A transmission geometry, the probed GHz waves have a wavevector $q = (4\pi/\lambda)\sin 45^{o} = 16.7\ \mu\text{m}^{-1}$, where $\lambda = 532\ nm$ is the laser wavelength. In the backscattering BLS, the probed wavevector $q = 4\pi n/\lambda$, where n is the refractive index of the material.

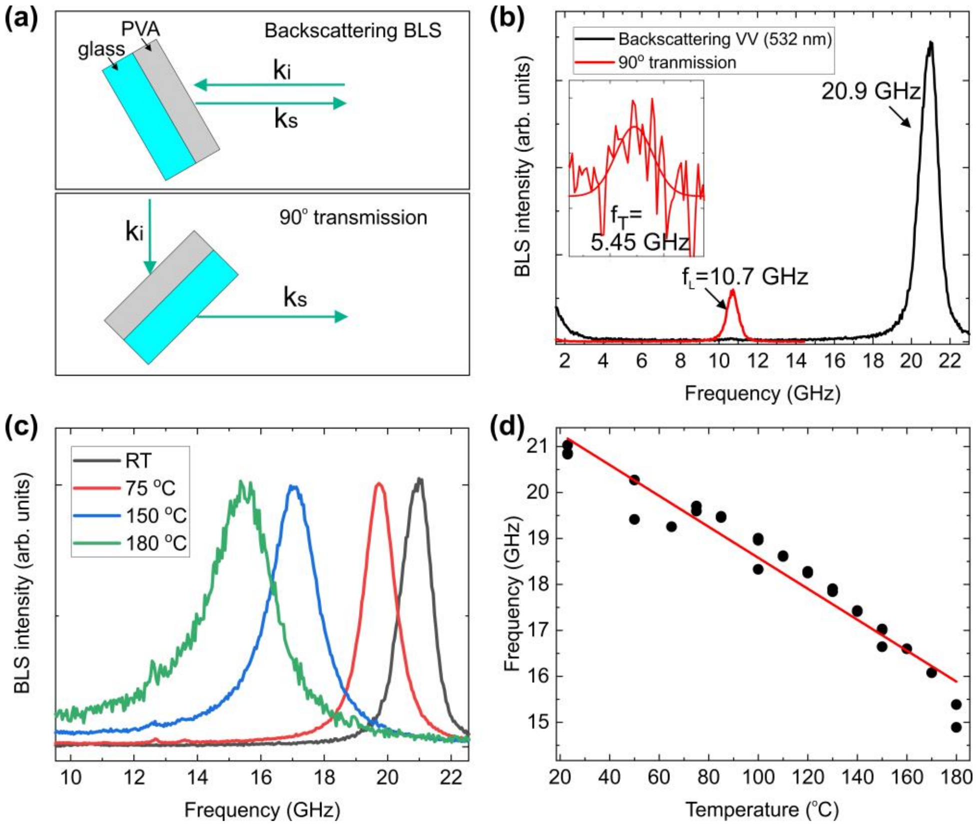

Figure S3: (a) Geometries for BLS. **(b)** BLS spectra in backscattering (black), 90 degrees transmission VV (red) and VH (inset). **(c)** Backscattering BLS at different temperatures. **(d)** The temperature dependence of the frequency measured in backscattering.

Based on the measured BLS spectra at room temperature (**Figure S3b**), the longitudinal and transverse sound velocities are C_L=4,025 m/s and C_T=2,050 m/s, respectively, the shear modulus is $G=p\cdot C_T^2$ =5 GPa, the Poisson ratio is μ=0.325, and the Young's modulus is $E=((1+v)\cdot(1-2v)/(1-v))\cdot p\cdot C_L^2$=13.25 GPa. The frequency of the longitudinal acoustic phonons, measured in backscattering, is strongly red-shifting as the temperature increases (**Figure S3c**),

moving from 20.9 GHz at RT, to ~15 GHz at 180 °C. The temperature-dependence of the frequency of the longitudinal acoustic waves in backscattering is shown in **Figure S3d**.

Fitting results with Lorentzian peak profiles:

For the BLS studies of the Au NRs in PVA, the spectra have been represented with Lorentzian peak profiles. The obtained peak parameters are summarized in Tables S1-S4 below.

Table S1: Peak parameters for the BLS spectra measured with 532 nm CW laser light (corresponding to upper panel in **Figure 3a** in the main article).

Parameters	Peaks					
Position (GHz)	6.65 ± 0.05	10.86 ± 0.02	17.13 ± 0.05	19.94 ± 0.05	28.86 ± 0.07	41.4 ± 0.1
Width (GHz)	2.1 ± 0.2	3.0 ± 0.2	2.5 ± 0.2	5.2 ± 0.2	8.9 ± 0.4	13 ± 1
Area ($\times 10^{-5}$ counts/mW/s)	19 ± 3	27 ± 2	14 ± 2	97 ± 5	67 ± 5	74 ± 10

Table S2: Peak parameters for the BLS spectra measured with 633 nm CW laser light (corresponding to lower panel in **Figure 3a** in the main article).

Parameters	Peaks						
Position (GHz)	7.3	11.5	18.0	23.2	27.6	31.4	39.9
Width (GHz)	3.3	2.4	4.2	4.7	1.4	9.7	10
Area ($\times 10^{-5}$ counts/mW/s)	335	100	50	68	18	62	80

Table S3: Peak parameters for the BLS spectra measured with 532 nm CW laser light (corresponding to upper panel in **Figure 3b** in the main article).

Parameters	Peaks					
Position (GHz)	6.0	11.3	17.3	24.8	28.0	37.6
Width (GHz)	5	5	3.7	4	6.8	11
Area ($\times 10^{-5}$ counts/mW/s)	16.8	12.2	15.6	11.2	10.0	13.6

Table S4: Peak parameters for the BLS spectra measured with 633 nm CW light (corresponding to lower panel in **Figure 3b** in the main article).

Parameters	Peaks					
Position (GHz)	8.2	11.8	15.8	27.6	30.0	39.7
Width (GHz)	4.3	4.1	1.9	1.1	29.6	21

Area ($\times10^{-5}$ counts/mW /s)	106	61	27.6	17.9	29.7	4.9

Plasmonic enhancement factors of BLS:

Table S5 below shows the plasmonic enhancement factor for the first two peaks of the BLS spectra in **Figure 3a** and **3b** in the main manuscript. The enhancement factor is defined as A^{633}/A^{532}, where A^{633} (A^{532}) is the area of the fitted Lorentzian peak. Since the frequencies of coupling vibrational modes are strongly affected by the absorbed laser light, which differs for 532 and 633 nm, the table gives the average frequency for the two spectra.

Table S5. BLS enhancement factors [I(633 nm)/I(532 nm)].

	Au700		Au825	
Average frequency (GHz)	7	11.5	7.2	11.4
Enhancement factor	11.7	7.2	6.3	5

High-frequency breathing mode for Au700 in PVA:

The BLS spectrum of Au700 NRs in PVA has a peak ~63 GHz (**Figure S4**). This frequency can be associated with the breathing NR mode. The frequency of the radial breathing mode (f_{br}) is given by:

$$f_{br}(n) = \frac{\tau_n}{2\pi R}\sqrt{\frac{E(1-v)}{p(1+v)(1-2v)}} \quad , \qquad (1)$$

where, v is the Poisson ratio, R the radius and τ_n the n-th root of the equation: $\tau J_0(\tau) = ((1-2v)/(1-v))J_1(\tau)$. Based on **Equation 1** the breathing mode appears at 64 GHz.

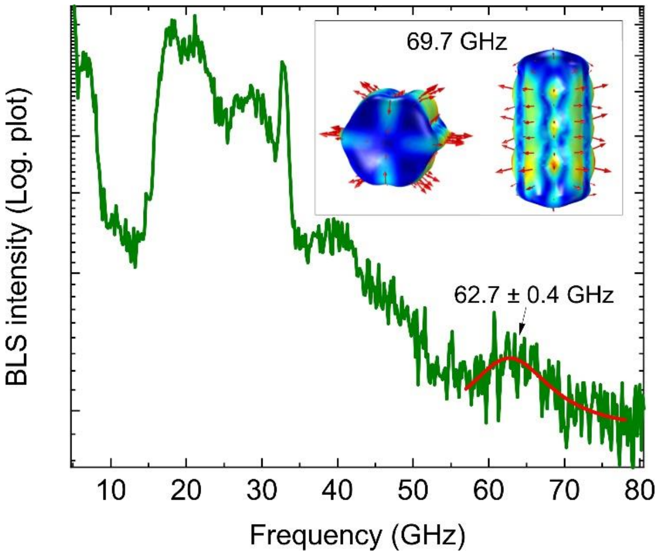

Figure S4: BLS spectrum up to 80 GHz of Au700 NRs in PVA measured with 532 nm light.

Laser-induced softening of coupled vibrational modes:

Compared to normal modes, coupled vibrational modes show a strong softening / frequency red-shift with increasing the incident laser power or equivalently the temperature. We have repeated the BLS measurements of **Figure 3** in the main article at various incident laser fluences with 532 nm and 633 nm laser light. **Figure S5a** shows the frequency of the first two lowest frequency peaks of Au700 as a function of the incident laser power for 532 nm (green points) and 633 nm (red points).

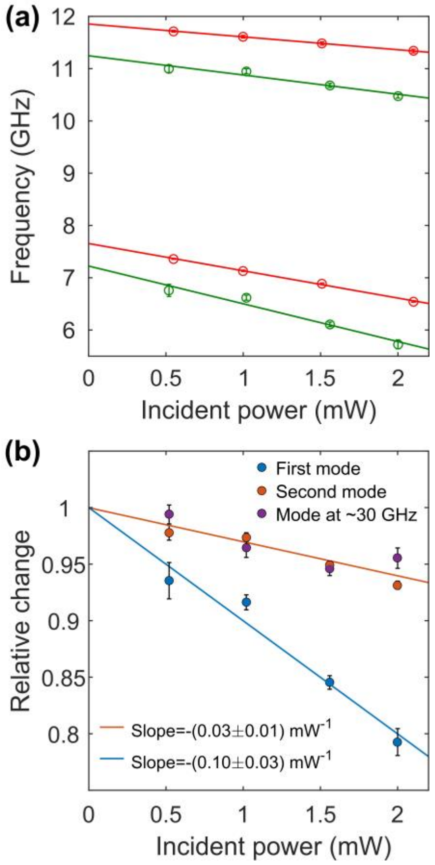

Figure S5: Laser-induced softening of Au nanorod vibrations. (a) The power dependent frequency of the first two BLS peaks of Au700 for 532 nm (green points) and 633 nm light (red points). The experimental points are fitted with linear functions (solid lines) to find the frequency at zero laser power. (b) The relative change of Au700 frequencies as a function of the incident laser power (λ=532 nm) for the mode at ~30 GHz and the two first peaks.

The linear representation of the two vibration frequencies versus incident laser power yields their values in the zero power limit and the rate of decrease with laser power. The similarity of the frequencies, 7.2 ± 0.3 GHz and 11.3 ± 0.2 GHz for 532 nm, and 7.66 ± 0.04 GHz and 11.85 ± 0.01 GHz for 633 nm suggests the first two peaks of the BLS spectra originate on the same type of vibration for both wavelengths. Noticeably, the plasmonic enhancement of BLS at 633 nm significantly reduces the experimental error compared to the spectra at 532 nm. Yet, the two frequencies display different dependence on the laser power, as shown in **Figure S5b**, with the lower frequency (~7.5 GHz) undergoing a stronger tendency for laser-induced softening, meaning a red-shift of the frequency. This behavior originates from the participation of the

surrounding soft matrix in the vibration and corroborates the notion that the first BLS peak corresponds to a coupling vibrational mode mediated by the PVA matrix.

The slope and the y-axis intercept for the linear fittings are shown in the table below.

Table S6:

	1st and 2nd peak at 532 nm		1st and 2nd peak at 633 nm	
Slope [mW^{-1}]	-0.10±0.03	-0.03±0.01	-0.073±0.004	-0.021±0.009
Intercept[GHz]	7.2 ± 0.3	11.3 ± 0.2	7.66 ± 0.04	11.85 ± 0.01

BLS of Au700 in PVA at a lower concentration, 0.3 vol%:

Figure S6 shows the BLS spectrum of Au700 NRs in PVA with 0.3 vol% recorded at 532 nm laser wavelength. This measurement was performed on a spot in the central region where the dispersion is dilute and semi-transparent. Due to the lower optical absorption, an incident laser power up to 10 mW without damaging the samples was utilized to increase the signal-to-noise ratio.

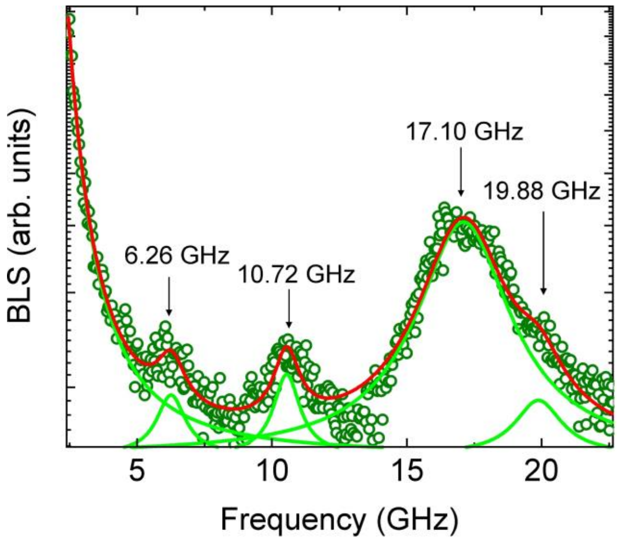

Figure S6: The BLS spectrum of Au700 in PVA at 0.3 vol% recorded with at 532 nm light wavelength in the central semi-transparent region of the film. The spot area has a diameter of about 2 μm while the film is several millimeters wide. The y-axis is in logarithmic scale and the experimental data (green circles) were represented with a sum (red curve) of four Lorentzian peaks (light green curves) plus a Rayleigh wing for the background. The peak at 17.1 GHz is assigned to the effective medium longitudinal phonon in the polymer nanocomposite.

The spectra are well represented by four Lorentzian peaks plus a Rayleigh wing for the background. In the 5-20 GHz range, all the spectral peaks of the Au700 NRs in PVA with 1 vol% (Fig. 3a) are also visible for the film at 0.3 vol%. Compared to the 1 vol% sample, the dilute Au700 sample displays a much stronger peak at 17 GHz due to the reduced light absorption and hence higher laser light intensity was used to record the spectrum of **Fig. S6**. This spectral peak is, therefore, assigned to the effective medium longitudinal phonon in the PVA nanocomposite.

BLS spectrum of Au800 with 532 nm light:

Figure S7 shows the normal modes and BLS spectra of polydisperse Au NRs in PVA with a concentration of 1 vol%. The average length (L) and diameter (D) of the NRs are 42.6 nm and 12.2 nm, respectively. Some examples of vibrational modes for isolated NRs of these size are shown in **Fig. S7(a)**. It is noted that for these NRs the frequency of the first extensional mode is expected to be very close to that of the effective medium acoustic phonons of the polymer matrix.

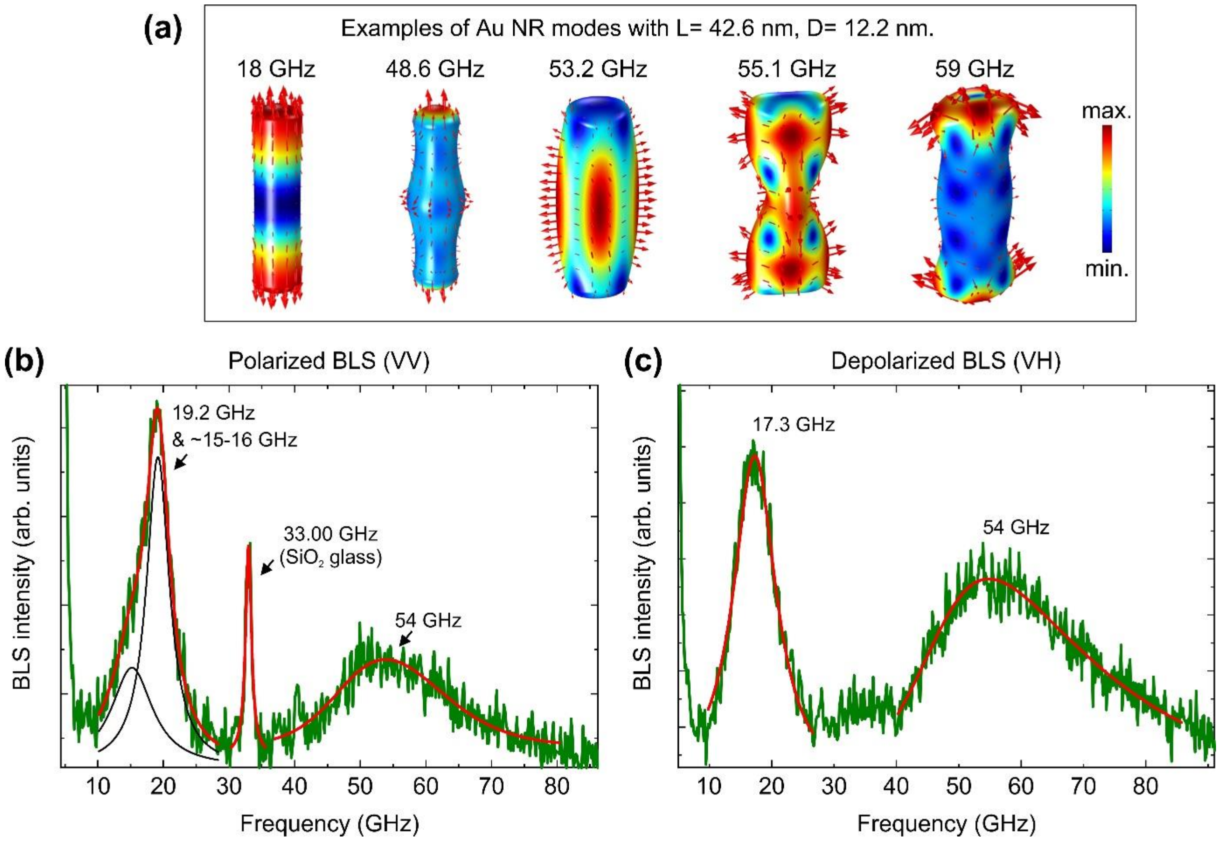

Figure S7: Normal modes and BLS spectra (532 nm light, 1 mW power) of Au NRs in PVA (1% per volume) with average length L=42.6 nm and average diameter D=12.2 nm. **(a)** Examples of nanorod vibrational modes for the monomer case. **(b)** BLS spectra in VV polarization 532 nm light and 1 mW incident power. **(c)** BLS spectra at the same conditions but with VH polarization. The experimental results are shown in green solid lines and the red line is a fit of the experiment data.

Figure S7(b) shows the BLS spectrum measured in VV polarization. In this case the collected light retains the polarization of the incident light, and gets inelastically scattered by the NRs as well as the PVA matrix and the underlying glass substrate. On the low frequency side, the spectrum contains a double Lorentzian peak with a weak and a strong component at ~15-16 GHz and at 19 GHz, respectively. The 19 GHz peak is attributed to the effective medium acoustic phonons of PVA. At higher frequencies, there is a broad asymmetric peak, which can be approximated by an asymmetric double Sigmoidal function (Asym2sig function of origin) with a maximum intensity at 54 GHz. The effective medium peak is suppressed in the depolarized (VH) BLS spectra shown in **Figure S7(c)**.

S3. Mechanical eigenmodes of Au NR monomers.

Au700 in air:

Figure S8 shows the normal modes of Au700 in air, taking into account the anisotropic elasticity of Au, as found with Comsol. The panels illustrate examples of bending, extensional, torsional and quadrupolar modes.

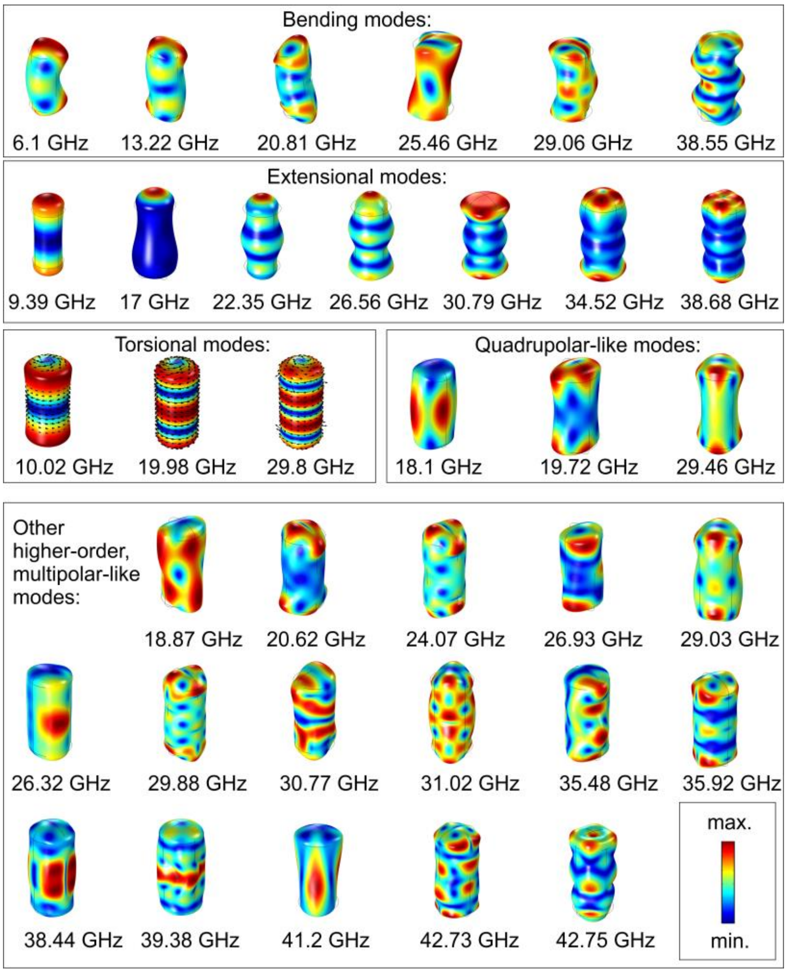

Figure S8: The complete set of Au700 normal modes in air up to 45 GHz.

Mechanical eigenmodes of Au825 in PVA:

Figure S9 below shows the normal modes of Au825 in PVA, which correspond to the optomechanical calculations of **Figure 4d** in the main article.

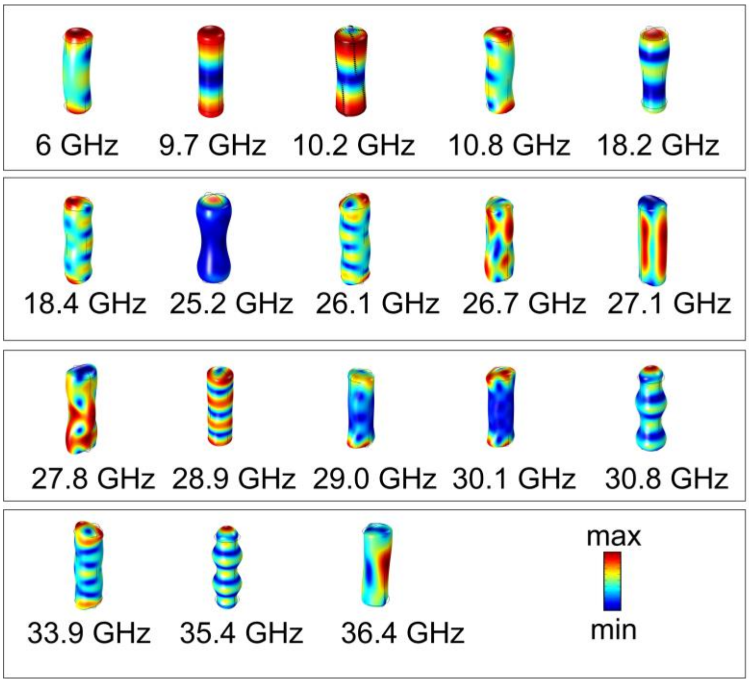

Figure S9: Mechanical eigenmodes of Au825 NRs between 5-40 GHz.

S4. Optomechanical calculations with 633 nm radiation for single Au700 and Au825 NRs.

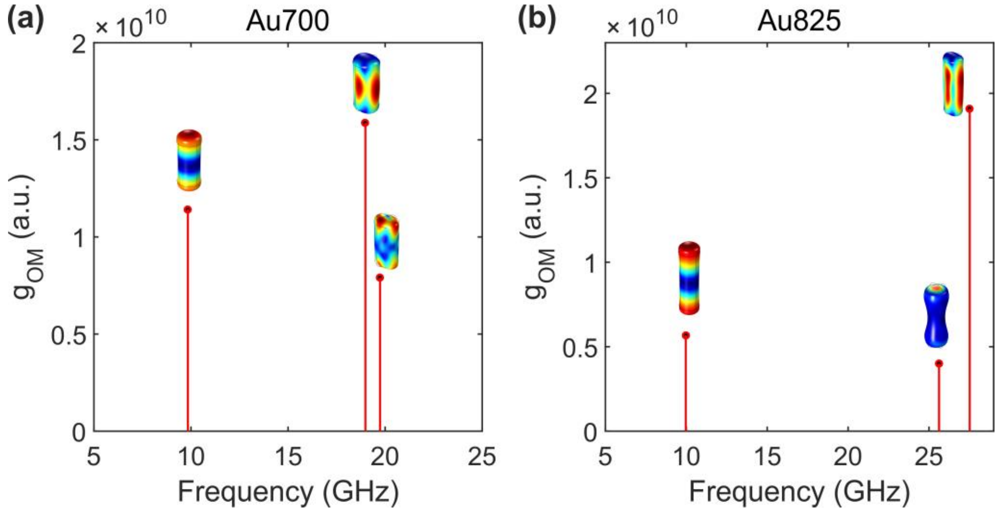

Figure S10: Optomechanical calculations with 633 nm radiation for single Au700 **(a)** and Au825 **(b)** NRs for the first three optomechanically active modes. The optomechanical coupling strengths have been averaged over x and z-polarizations.

S5. Extinction spectra of Au825 in PVA.

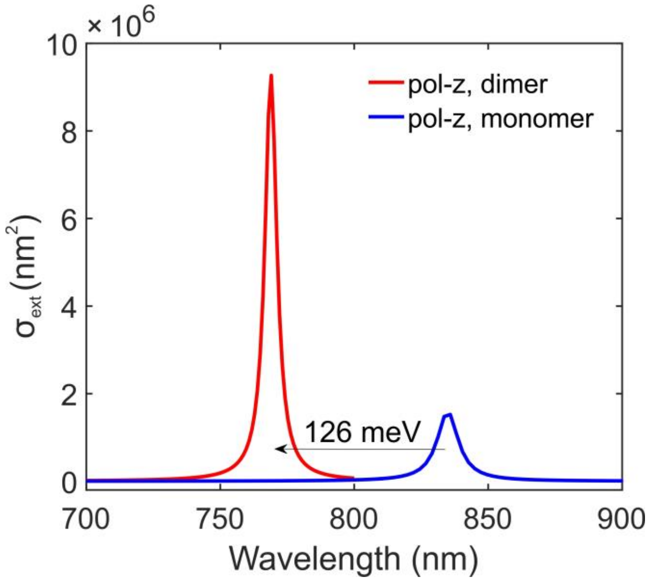

Figure S11: The extinction spectra of the longitudinal plasmon resonances for monomers and dimers of Au825 (2.5 nm gap).

S6. Absorption spectrum of Au800 NRs

Figure S12 shows the measured absorption spectrum of Au800 NRs in PVA (blue circles) together with the FEM calculated spectrum (blue dashed-dot line). The longitudinal plasmon is located at ~800 nm in both theory and in the measurement. In the theory, the transverse plasmon resonance gives a single peak at ~520 nm. However, the experiment is better represented by a double Gaussian peak (red solid line) with two components, at 520 nm and at approximately 600 nm. The latter peak can be attributed to coupled transverse plasmons in Au800 dimers.

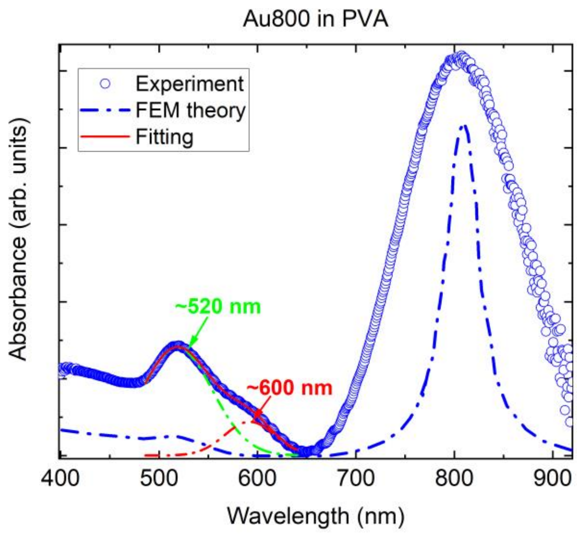

Figure S12: Experimental absorption spectrum of Au800 NRs in PVA (blue circles) together with the FEM calculated spectrum (blue dashed-dot line). The transverse resonance peak can be represented with a double Gaussian fit (red line) with components at ~520 nm (green dashed-dot) and ~600 nm (red dashed-dot). The latter is due to plasmonic coupling in Au800 NR aggregates.

S7. Comparison of BLS enhancement with PVA and PVP for Au800

The emergence of new BLS peaks that has been observed for Au800 NRs in PVA (**Figure 3c** in the main article), when switching the wavelength of light from 532 nm to 633 nm, is also visible for Au800 NRs in PVP (**Figure S13**). The PVP peak appears at 11.9 GHz for 633 nm light and at 14.7 GHz for 532 nm light due to the different wavevectors of the bulk phonons probed with BLS. The same shift occurs for the peak originated from the glass substrate. This behavior cannot explain the rest of the differences that arise due to the existence of Au800 NRs. In particular there is a strong BLS peak, observed only with 633 nm, which appears at 37 GHz for PVP and ~40 GHz for PVA. The observed red-shift is expected since the PVP is softer than PVA. For PVP, C_L = 2,989 m/s, C_T = 1,443 m/s, and ρ_{PVP} = 1200 kg/m^3, where C_L and C_T are the longitudinal and transverse sound velocity, respectively (see Reference 52 in the main article and **Section S3** for a comparison with PVA).

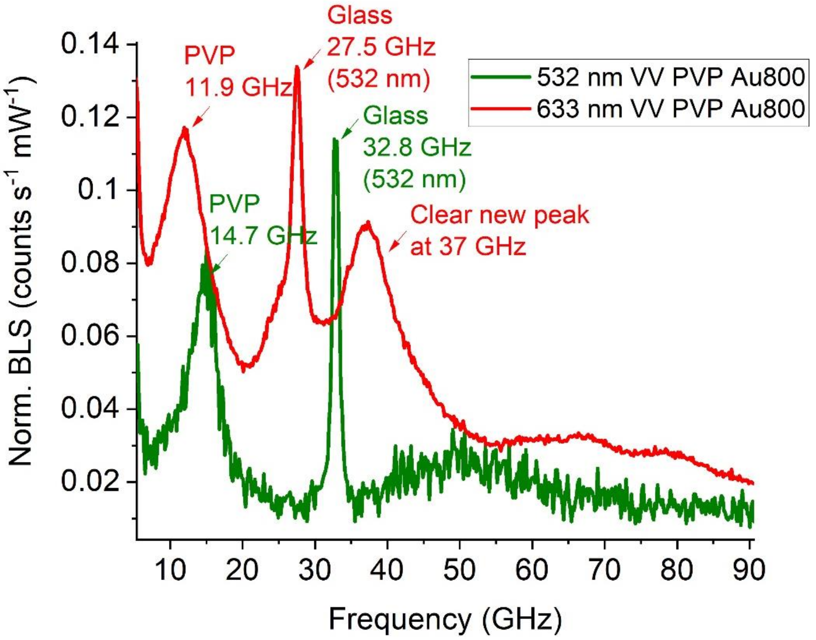

Figure S13: Normalized BLS spectra of Au800 NRs in PVP, on the coffee ring, with 532 nm (green curve) and 633 nm (red curve) light. The measurements were carried with VV polarization.

S8. Additional measurements and temperature effect on BLS spectra of Au800 – PVA nanocomposite.

The BLS spectra of Au800 NRs have been measured with both 633 nm (**Figure S14**) and 532 nm (**Figure S15**) wavelength of light in VV and VH polarization. **Figure S15** shows BLS measurements of the effective medium frequency and velocity in backscattering geometry for Au800 in PVA (1% vol.) in VV polarization. These measurements were carried on the coffee ring with 1-8 mW incident power of 532 nm light. The effective medium peak (see also **Figure S7(b)**) is asymmetrically broaden towards lower frequencies due to the presence of Au800 NRs. In **Figure S15** the effective medium peak is represented with double Lorentzian functions (as in **Figure S7(b)**) in order to extract the position of maximum intensity. In backscattering the wavenumber is given by $Q=4\pi n/\lambda$, where λ=532 nm and n=1.4881, and the sound velocity is

C_L=2πf/Q. In the limit of zero incident laser power, the C_L=3600±50 m/s. The velocity is decreasing by ~110 m/s for every 1 mW of incident 532 nm light.

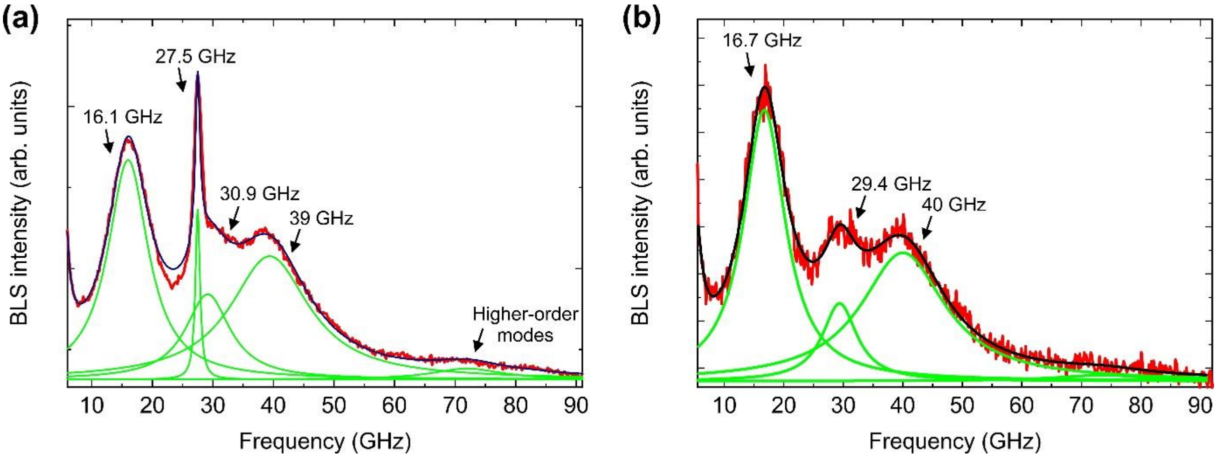

Figure S14: BLS measurements (633 nm light, 1 mW power) of Au800 NRs in PVA (1% per volume, with L= 42.6 nm and D= 12.2 nm) in VV (a) and VH (b) polarization.

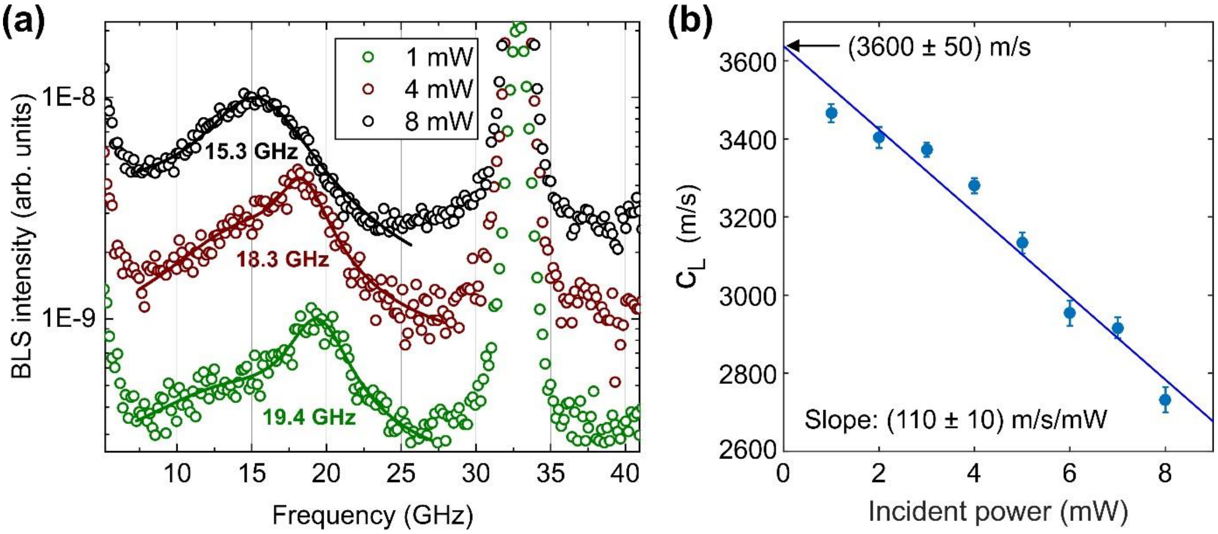

Figure S15: (a) Experimental backscattering BSL spectra of 1% Au800 in PVA in VV polarization. **(b)** The incident power dependence of the velocity of longitudinal phonons of the effective medium.

S9. Model of optomechanical coupling

Selection of the model and underlying theory: The reformulation of Maxwell's equation as an eigenvalue problem is an important theoretical tool for studying photonic metamaterials, *e.g.*, photonic crystal slabs and photonic crystal fibers. In this formalism, the spatial distribution of the magnetic field intensity $\vec{H}(\vec{r})$ obeys $\hat{\Theta}\vec{H}(\vec{r}) = (\omega/c)^2\vec{H}(\vec{r})$, where $\hat{\Theta} = \vec{\nabla} \times [(1/\varepsilon(\vec{r}))\vec{\nabla} \times]$ is a Hermitian operator. The eigenvalue problem for the electric field

distribution ($\vec{E}(\vec{r})$) can be extracted with the relationship $\vec{E}(\vec{r}) = (-1/i\omega\varepsilon_o\varepsilon(\vec{r}))\vec{\nabla} \times \vec{H}(\vec{r})$. In the presence of translational symmetry, the assumed solutions are Bloch states, leading to the calculation of the photonic band structures. The formulation of electromagnetism as an eigenvalue problem paves the way for applying perturbation theory, in order to find the effect of small perturbations on the photonic states.

When the interactions involve optical and mechanical degrees of freedom, the total optomechanical (OM) coupling rate $g_{OM} = g_{PE} + g_{MI}$, where g_{PE} is the coupling rate of the photoelastic (PE) effect in the bulk, and g_{MI} is the coupling rate of the moving interface (MI) effect on the surface. Based on first order perturbation theory – see Reference 48 of the main article – one gets:

$$g_{PE} = -\frac{\omega}{2}\frac{\langle E|\delta\varepsilon|E\rangle}{\iiint \vec{E}\cdot\vec{D}dV}\sqrt{\frac{\hbar}{2M_{eff}\Omega}} \quad , \quad (2)$$

where ω and Ω are the optical and acoustic angular frequencies, respectively, $\iiint \vec{E}\cdot\vec{D}dV$ is a normalization factor for the total electromagnetic energy stored in the system, $M_{eff} = \iiint \rho \left\|\vec{U}\right\|^2 dV$ is the effective mass, and $\sqrt{\hbar/2M_{eff}\Omega}$ is the zero-point motion of the mechanical oscillator. For the moving interface contribution the coupling rate is given:

$$g_{MI} = -\frac{\omega}{2}\frac{\iint(\vec{U}\cdot\hat{n})(\Delta\varepsilon\vec{E}_{\parallel}^2 - \Delta\varepsilon^{-1}\vec{D}_{\perp}^2)d\vec{S}}{\iiint \vec{E}\cdot\vec{D}dV}\sqrt{\frac{\hbar}{2M_{eff}\Omega}} \quad , \quad (3)$$

where $\vec{U} = \vec{u}/\max(|\vec{u}|)$ is the normalized mechanical displacement field, $\hat{n}$ is the unitary vector normal to the surface, and $\Delta\varepsilon = \varepsilon_1 - \varepsilon_2$ is the difference of the dielectric permittivities at the material-air interface. In a previous work, relationships (2) and (3) provided a satisfactory description of the BLS spectra of Ag nanospheres dispersed in polymers measured with a single laser wavelength (532 nm) (Reference 52 of the main article).

Nevertheless, the conventional first order perturbation theory is not fully compatible with plasmonic, dissipative systems, since it requires a purely real and positive dielectric function $\varepsilon(\vec{r})$. As a matter of fact, the operator $\hat{\Theta}$ is not Hermitian in this case and hence it is not obvious to define an internal product $\langle E|\delta\varepsilon|E\rangle$ where $\langle E|$ is simply the complex conjugate of $|E\rangle$. A more general approach has been proposed lately based on quasi-normal eigen modes (QNMs) well adapted for open and dissipative systems like plasmonic resonators [49,50]. However, such methods require the computation of the quasi-normal eigenmodes of the considered plasmonic system [51]. Furthermore, in our case we intend to determine the optomechanical coupling rate (OM) at two specific lasers wavelengths (532 and 633 nm), which do not necessarily correspond to any quasi-eigenmode of the NRs. In that regard, we opted rather for the computation of the optical force F_{op} per unit energy based on the moving boundary and the photoelastic effects, which is proportional to the OM intensity as $F_{op} \propto g_{OM}$ (Ref. 36 in main article). We calculated the two overlap integrals involving the superposition of the electromagnetic and mechanical fields for these effects and took their sum $(\iint f_{MB}\cdot dS + \iiint f_{PE}\cdot dV) = F_{op}$, representing the induced optomechanical force (per unit energy). The latter is proportional to the single-photon optomechanical coupling rate as (Refs 36,37 in main article):

$$g_{OM} = -\frac{\omega x_{zpf}}{2}F_{op}\,. \qquad (4)$$

The reason for this choice is that in the expression of F_{op} it is the scattered/incoming field which is used instead of the QNMs as shown by Equations (6) and (7) in the main article. Employing the scattered/incoming field presents the advantage of choosing freely any wavelength of the incoming field (i.e., 532 and 633 nm) to compute the optical force F_{op} (and hence $g_{OM} \propto F_{op}$).

Geometry model configuration of Au NRs monomer/dimer in PVA matrix:

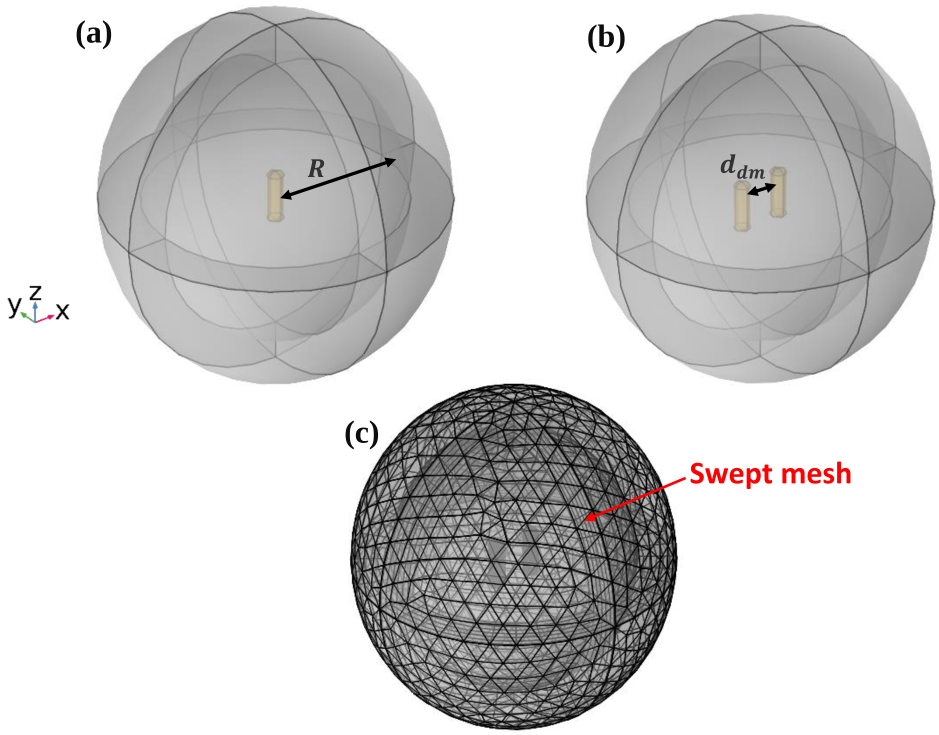

Figure S16: Sketch of the employed 3D model to determine Au NRs mechanical eigen-vibrations modes in spherically shaped PVA matrix, which is surrounded by perfectly matched layers (PMLs) in order to get rid of unwanted reflections (a) Single Au NR (a) and dimer at an interparticle separation d_{dmr} (b)**.** (c) Example of the applied mesh in case of Au NRs dimer.

In order to get the mechanical eigen-vibrations modes of a single NR or a dimer, we employ the geometry models depicted in Figs. S12 (a) and (b) consisting in cylindrically shaped single/dimer nanoparticle (s) set symmetrically at the core of a spherically shaped PVA material. The latter represents the matrix where particles are embedded; we set perfect matched layers all around the PVA sphere to truncate the model which, enables to prevent perturbing reflections. It should be pointed out that the PVA sphere size is such that its radius is as, $R \gtrsim \lambda_0/2$, where $\lambda_0 \sim \sqrt{E_{\text{PVA}}(1-\nu_{PVA})/\rho(1+\nu_{PVA})(1-2\nu_{PVA})}/f_0$ is roughly the wavelength of longitudinal waves propagating in the matrix. In this formula, ν_{PVA}, ρ and f_0 are the Poisson's ratio, density of PVA and the wave frequency, respectively. This approximate size set of the sphere is important so that the wavelengths of longitudinal waves in the PVA matrix (induced by the NR vibrations) get correctly absorbed by the PML without reflection. Note that the transverse waves wavelength is smaller meaning, its value is already adapted to the sphere size once we fulfill the condition $R \gtrsim \lambda_0/2$. Regarding the PMLs thickness l_{PML}, we set a value of

l_{PML}~$\lambda_0/5$, where, numerical simulations show that outgoing waves are well absorbed by PMLs.

As concerns the applied mesh, we apply the following scheme to assure numerical convergence: i) inside the NR we set $\Delta = R_{NR}/4$ i.e., a minimum of four points per particle radius is chosen, ii) in the PVA sphere we apply a finer mesh and iii) across the PMLs we set a swept mesh with six points. In **Fig. S16c**, we show an example of the applied mesh in case of a dimer. Note that we use tetrahedral type mesh finite elements. For the optomechanical computations, we employ the aforementioned model using both solid mechanical and electromagnetic waves modules. In particular, we compute first the mechanical eigenmodes in an eigenfrequency study step then while storing the results we sequentially compute the scattered electric field. Thereafter, the relevant integrals giving the optical force F_{op} are computed. Finally, the modeled geometry of a Au700 NR (yellow lines), overlapped over a real TEM image, is shown in **Figure S17**. The Au NRs are simulated as two identical ellipsoids connected by a cylinder. The ellipsoids have radii $R_1=R_2=17.85$ nm and $R_3=8.925$ nm. The cylinder has radius R=R1 and length L=82.1 nm-2R_3. The long Au NR axis (length) is parallel to the [001] crystallographic direction and the z-axis of the simulation (**Figure S16**). Thus the anisotropic properties are always described with the elasticity matrix given in the main text, as the Au NRs are single-crystalline based on TEM measurements.

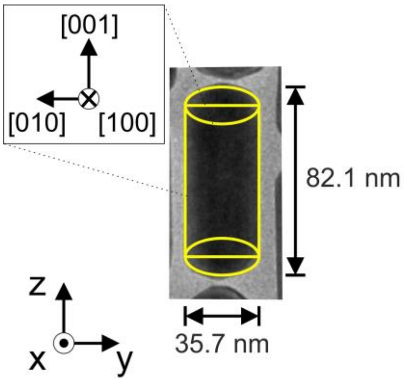

Figure S17: The geometry of Au700 used in the plasmonic, mechanical and optomechanical simulations of Figures 4 and 5 in the main article.

Through all the above we achieved an adequate agreement between the frequencies of the OM-active modes and the observed BLS peaks for the three types of Au NR monomers (**Figure 4** in main article). When applied to Au NR dimers, this model provides an explanation for the BLS activity of bending modes and the BLS enhancement with off-resonant 633 nm light (**Figures 5** and **6** in main article).
